# A predictive propensity measure to enter REM sleep

**DOI:** 10.3389/fnins.2024.1431407

**Published:** 2024-08-30

**Authors:** Alexander G. Ginsberg, Madelyn Esther C. Cruz, Franz Weber, Victoria Booth, Cecilia G. Diniz Behn

**Affiliations:** ^1^Department of Mathematics, University of Utah, Salt Lake City, UT, United States; ^2^Department of Mathematics, University of Michigan, Ann Arbor, MI, United States; ^3^Department of Neuroscience, University of Pennsylvania, Philadelphia, PA, United States; ^4^Department of Anesthesiology, University of Michigan, Ann Arbor, MI, United States; ^5^Department of Applied Mathematics and Statistics, Colorado School of Mines, Golden, CO, United States; ^6^Department of Pediatrics, University of Colorado Anschutz Medical Campus, Aurora, CO, United States

**Keywords:** sleep cycle, NREM-REM cycles, ultradian rhythms, sequential REM episodes, REM pressure, hourglass process

## Abstract

**Introduction:**

During sleep periods, most mammals alternate multiple times between rapid-eye-movement (REM) sleep and non-REM (NREM) sleep. A common theory proposes that these transitions are governed by an “hourglass-like” homeostatic need to enter REM sleep that accumulates during the inter-REM interval and partially discharges during REM sleep. However, markers or mechanisms for REM homeostatic pressure remain undetermined. Recently, an analysis of sleep in mice demonstrated that the cumulative distribution function (CDF) of the amount of NREM sleep between REM bouts correlates with REM bout duration, suggesting that time in NREM sleep influences REM sleep need. Here, we build on those results and construct a predictive measure for the propensity to enter REM sleep as a function of time in NREM sleep since the previous REM episode.

**Methods:**

The REM propensity measure is precisely defined as the probability to enter REM sleep before the accumulation of an additional pre-specified amount of NREM sleep.

**Results:**

Analyzing spontaneous sleep in mice, we find that, as NREM sleep accumulates between REM bouts, the REM propensity exhibits a peak value and then decays to zero with further NREM accumulation. We show that the REM propensity at REM onset predicts features of the subsequent REM bout under certain conditions. Specifically, during the light phase and for REM propensities occurring before the peak in propensity, the REM propensity at REM onset is correlated with REM bout duration, and with the probability of the occurrence of a short REM cycle called a sequential REM cycle. Further, we also find that proportionally more REM sleep occurs during sequential REM cycles, supporting a correlation between high values of our REM propensity measure and high REM sleep need.

**Discussion:**

These results support the theory that a homeostatic need to enter REM sleep accrues during NREM sleep, but only for a limited range of NREM sleep accumulation.

## 1 Introduction

Rapid-eye-movement (REM) sleep in mammals alternates with non-REM (NREM) sleep to create irregular cycles during sleep periods (Le Bon, 2021). Current theory proposes that an “hourglass”-type homeostatic drive promotes transitions into REM sleep (Benington et al., 1994; Vivaldi et al., 1994). That is, the drive for REM sleep builds up between REM bouts, specifically during NREM sleep (Benington et al., 1994; Heller, 2021; Park et al., 2021), and partially discharges during REM sleep. This theory then predicts correctly that NREM bouts have longer durations when they follow longer REM bouts (Benington and Heller, 1994; Vivaldi et al., 1994, 2005; Barbato and Wehr, 1998; Park et al., 2021; Cajochen et al., 2023), presumably because more of the drive for REM sleep is discharged during the REM episode. However, the length of a NREM bout is not associated with the duration of the subsequent REM bout (Benington and Heller, 1994; Park et al., 2021), so it is unclear how the propensity for REM sleep builds up during NREM sleep. Answering this requires a predictive measure for the need to enter REM sleep, i.e., a measure of REM propensity.

Various measures of the propensity to enter REM sleep have been proposed. One such measure is the total time in REM sleep during a fixed interval of time [e.g., 30-min intervals within a longer sleep episode (Chang et al., 2015) or a complete nighttime sleep episode (Nielsen et al., 2010)]. Additionally, the density of NREM to REM transitions during sleep has been proposed to reflect the need to enter REM sleep (Benington and Heller, 1994; Bassi et al., 2009). Sleep latency is a measure of general sleepiness (Dijk et al., 2010), and an analogous measure for REM sleep propensity would be latency to REM sleep from the end of the preceding REM bout. However, these measures provide descriptors for the occurrence of REM sleep rather than a prediction for the timing of REM sleep onset.

Conceptually, the propensity for REM sleep should reflect the probability of entering REM sleep at a certain time during a sleep episode. Such a probability can be computed from sleep recordings as was recently done by Park et al. (2021). They propose a data-driven, probabilistic measure of propensity for REM sleep: the cumulative distribution function (CDF) of the amount of NREM sleep between REM bouts. Analyzing spontaneous sleep data in mice, Park et al. (2021) found that the CDF of the amount of NREM sleep between REM bouts was predictive for the duration of REM episodes. However, by definition, this CDF does not give the likelihood *of entering REM sleep in the future*. Instead, the CDF evaluated at some value *t* gives the probability that REM sleep *has already been entered* after *t* seconds of NREM sleep. Further, interpreting the CDF as a REM propensity measure assumes that the REM propensity shares the properties of the CDF: specifically, the REM propensity increases with time in NREM sleep and is thus an hourglass process. As a result, the CDF cannot be used to test whether a REM propensity measure represents an hourglass process.

In this paper, we build on the work of Park et al. (2021) and, we propose an alternative REM propensity measure that is defined precisely as the probability of entering REM sleep in the near future. We apply this measure to investigate the hypothesis that the need for REM sleep accumulates during NREM sleep. Analyzing spontaneous sleep data in mice (Park et al., 2021), our results show that, during the light phase only, this REM sleep propensity measure is significantly correlated with features of the subsequent REM bout. In particular, the propensity is positively correlated with the duration of the subsequent REM bout. Further, higher propensities following longer REM bouts, called “single REM bouts” (Zamboni et al., 1999; Park et al., 2021), are correlated with a higher probability of being followed by short REM bouts, called “sequential REM bouts” (Kripke et al., 1968; Ursin, 1970; Merica and Gaillard, 1991; Amici et al., 1994; Zamboni et al., 1999; Gregory and Cabeza, 2002; Park et al., 2021) that occur in sequences of short REM/NREM alternations. However, we also find that for mice, as the amount of time spent in NREM sleep increases, this REM propensity measure increases until it reaches a peak value; REM propensity eventually decays to zero as the time spent in NREM sleep continues to increase. Our analysis shows that after the propensity reaches its peak value, it ceases to be significantly correlated with the above features of the subsequent REM bout. This suggests that the amount of time in NREM sleep drives transitions into REM sleep and acts like an hourglass process, but only for a limited range of NREM sleep accumulation.

## 2 Results

We define our proposed REM propensity measure as follows. At a particular time, let |*N*| represent the total amount of NREM sleep that has occurred since the last REM bout (ignoring time spent in wake). We propose that the propensity to enter REM sleep at that time is the probability that the transition to REM sleep occurs before the amount of NREM sleep has increased by another Δ|*N*| seconds. This probability is defined as:


(1) $$\begin{array}{ll} p_{\text{Δ}|N|}\left(\left|N\right|\right) & =\frac{CDF\left(\left|N|+\text{Δ}|N\right|\right)-CDF\left(\left|N\right|\right)}{1-CDF\left(\left|N\right|\right)}. \end{array}$$

It can be verified by Bayes' law that *p*_Δ|*N*|_(|*N*|) in Equation 1 is indeed the desired probability (see Supplementary material S1). For this propensity measure to reflect an “hourglass-like” process, it should increase with the amount of NREM sleep accumulation: i.e., *p*_Δ|*N*|_(|*N*|) increases with |*N*|. We derive conditions that determine whether *p*_Δ|*N*|_(|*N*|) increases with |*N*| in Supplementary material S2.

**Figure 1:**
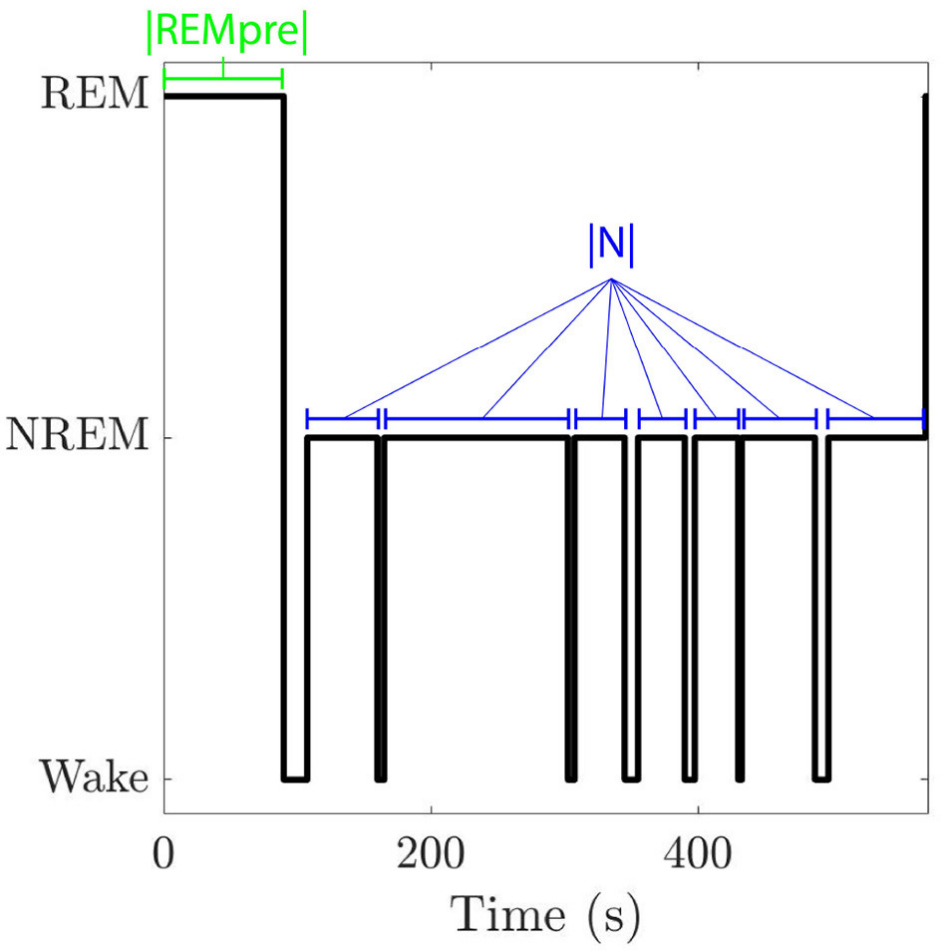
Hypnogram for one REM cycle. A REM cycle consists of an initial bout of REM sleep followed by an inter-REM interval where both NREM sleep and wake may occur. We denote the length of the cycle's initial REM bout by |*REMpre*| and the total duration of NREM sleep that occurs in the cycle's inter-REM interval by |*N*|.

We compute this REM propensity measure from data in Park et al. (2021), which contains 125 recordings from 72 mice housed in 12:12 h light and dark conditions with unlimited access to food and water. REM, NREM, and wake states were scored in 2.5 s bins during spontaneous behavior in the light (7 a.m.–7 p.m.) and dark (7 p.m.–7 a.m.) phases (see Park et al., 2021 for more details about the recordings). In each phase, we identify “REM cycles” (Figure 1), defined as the period between the start of one REM bout and the beginning of the next REM bout (Kripke et al., 1968; Ursin, 1970; Merica and Gaillard, 1991; Park et al., 2021).

**Figure 2:**
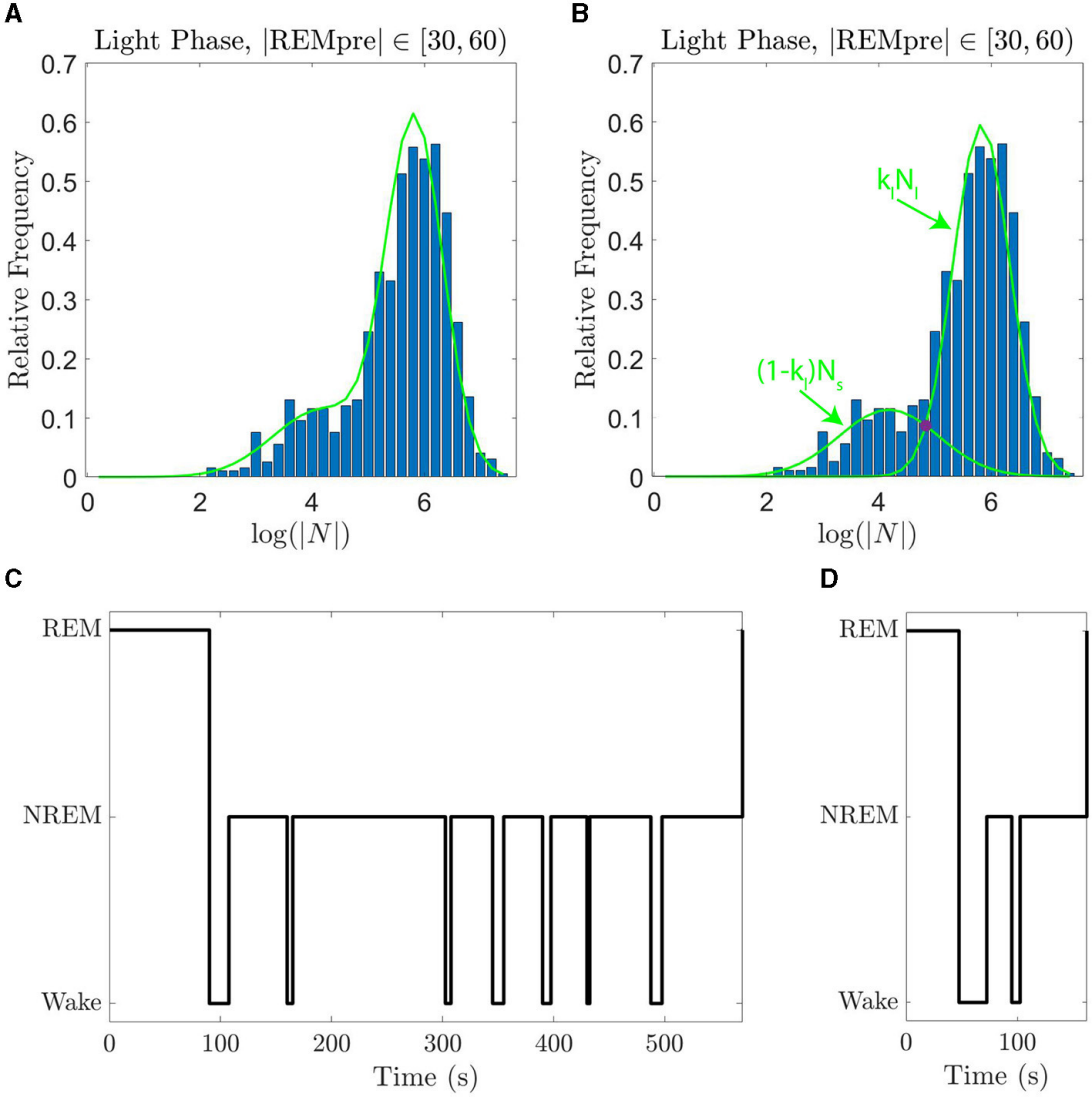
GMMs fit the data well and can be used to distinguish single and sequential REM episodes. **(A)** The empirical PDF of log(|*N*|) (blue histogram) along with the GMM fit curve (green line) for log(|*N*|) for REM cycles falling during the light phase with REMpre ∈[30, 60) s. **(B)** The empirical PDF of log(|*N*|) (blue histogram) with the normal distributions *N*_*l*_ and *N*_*s*_ that comprise the GMM when summed with weighting *k*_*l*_ (green curves). The value of |*N*| at which *k*_*l*_*N*_*l*_ and (1−*k*_*l*_)*N*_*s*_ intersect (purple circle) is the cutoff below which we label REM cycles as “sequential”, and above which we label REM cycles as single. **(C)** An example of a single REM cycle. **(D)** An example of a sequential REM cycle. Sequential REMs tend to have shorter |*REMpre*| and |*N*|.

The total duration of a REM cycle between a preceding REM bout, called REMpre, and the subsequent REM bout, called REMpost, includes the REMpre duration, denoted as |*REMpre*|, the cumulative duration of all NREM sleep during the inter-REM interval, denoted by |*N*|, and the cumulative duration of any wake occurring during the inter-REM interval (Figure 1). Following the analysis in Park et al. (2021), we separate REM cycles into bins based on REMpre durations. The bins are [0, 30), [30, 60), [60, 90), [90, 120), [120, 150), [150, 180) s, and |*REMpre*| >180 s. For each |*REMpre*| bin, we analyze REM cycles to identify the |*N*| values. Then we fit the distribution of log(|*N*|) values to a Gaussian Mixture Model (GMM), described by:


$$\begin{array}{ll} {\mathbb{P}}_{GMM}\left(x\right) & =k_{l}N_{l}\left(x\right)+\left(1-k_{l}\right)N_{s}\left(x\right), \end{array}$$

where *N*_*s*_ and *N*_*l*_ are normal distributions with means given, respectively, by μ_*s*_ and μ_*l*_ where μ_*l*_>μ_*s*_, and standard deviations given, respectively, by σ_*s*_ and σ_*l*_. Further, *k*_*l*_∈[0, 1] is a weighting parameter that describes the relative contributions of each distribution; *k*_*l*_ = 1 indicates that only the distribution with the larger mean contributes to the GMM. We show in Figures 2A, B an example of the GMM fit to log(|*N*|) data for |*REMpre*| in [30, 60) s and the weighted normal distributions *k*_*l*_*N*_*l*_ and (1−*k*_*l*_)*N*_*s*_ which comprise it.

As introduced in Park et al. (2021), the two modes of each GMM fit capture “sequential” REM cycles and “single” REM cycles (Figure 2). Specifically, the *N*_*s*_ portion of the GMM corresponds to sequential cycles, for which |*N*| tends to be smaller; whereas the *N*_*l*_ portion of the GMM corresponds to single cycles, for which |*N*| tends to be larger. The GMM fit for each |*REMpre*| bin qualitatively matches the PDF of log(|*N*|) for the bin (Supplementary Figure S1). Furthermore, using corrected Kolmogorov-Smirnov tests (Section 4.2), we find that the GMM generally quantitatively matches the distribution of log(|*N*|) values. Namely, for each bin for light and dark period data with one exception, we fail to reject the null hypothesis that the distribution of log(|*N*|) is truly the GMM we have identified. For the |*REMpre*|∈[60, 90) s for light period data, we did not fail to reject the null hypothesis. We report the GMM parameters (*k*_*l*_, μ_*l*_, σ_*l*_, μ_*s*_, and σ_*s*_) for the fits for each |*REMpre*| bin in Supplementary Figure S2.

### 2.1 REM propensity exhibits a peak after which it decays to zero

From the GMM fits of the data, we compute the CDF describing the probability that the mouse enters REM sleep after |*N*| s of NREM sleep since the end of the last REM bout and use that CDF directly in Equation 1. Specifically, for Δ|*N*| = 30 seconds of NREM, i.e., the “near future”, we compute the propensity for the mouse to re-enter REM as:


(2) $$\begin{array}{ll} p_{30}\left(\left|N\right|\right) & =\frac{CDF\left(|N|+30\right)-CDF\left(\left|N\right|\right)}{1-CDF\left(\left|N\right|\right)}. \end{array}$$

Thus, *p*_30_(|*N*|) represents the probability that when the mouse has accumulated |*N*| s of NREM sleep since the last REM episode (without re-entering REM), the mouse will re-enter REM before it has undergone 30 s more of NREM sleep. We show some validation studies of this propensity measure in Section 4.3.

**Figure 3:**
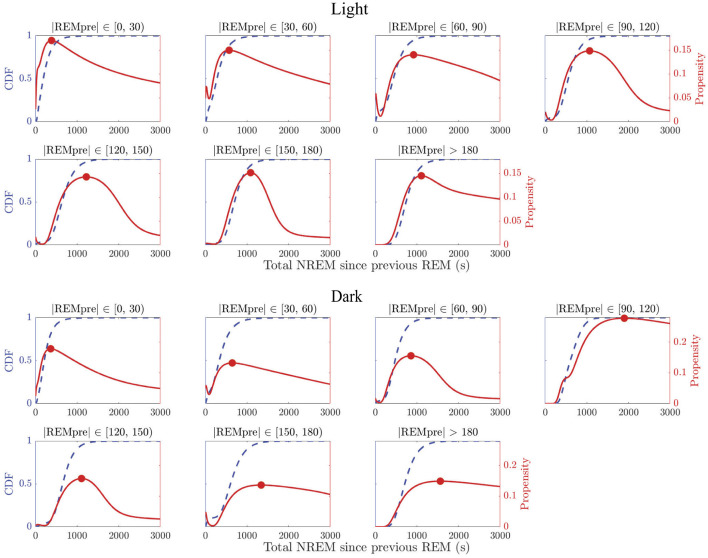
REM propensity measures as a function of NREM sleep duration. Panels show REM propensities (red curves) for REM cycle data in each |*REMpre*| bin, in the light (top 2 rows) and dark (bottom 2 rows) phases. For comparison, the CDF (adjusted from log(|*N*|) to |*N*|) derived from the GMM fit for each of the binned data is also displayed (blue dashed curves). The REM propensity *p*_30_(|*N*|) was calculated utilizing Equation 2. Each propensity curve has a local maximum (red filled circle) between 500 and 1,500 s, and decays to 0 as |*N*| → ∞.

We find that the propensity *p*_30_(|*N*|) does not increase monotonically for any |*REMpre*| bin in light or dark periods (Figure 3, red curves). By contrast, for each |*REMpre*| bin, the propensities are non-monotonic with at least one local maximum from which the propensity decays to zero as |*N*| → ∞. In fact, we can show that any propensity defined as in Equation 1 for which the CDF of the log of the underlying accumulating quantity is a GMM must have at least one local maximum and must eventually decay to zero (see Supplementary material S3 for details). This decrease in the REM propensity for large |*N*| suggests that in long REM cycles, the amount of NREM sleep alone may cease driving an hourglass-like need to transition to REM sleep.

We also find that the propensity reflects the presence of sequential REM bouts. In particular, in the data for shorter |*REMpre*| values, which we expect to contain more sequential REM cycles, we find that the propensity often initially decreases to a local minimum value (see e.g., |*REMpre*| bins [30, 60) and [60, 90) s in Figure 3 for both the light and dark periods) or shows a transient decrease in the rate at which it grows (see e.g., |*REMpre*| bins [0, 30) s for both the light and dark periods). This reflects the presence of cycles associated with both sequential and single REM bouts in these bins. Specifically, initially higher propensity values that then decrease reflect the presence of sequential REM cycles at shortest |*N*| values and their absence at slightly longer |*N*| values. The eventual subsequent increase in propensity to the local maximum reflects the occurrence of single REM cycles with longer |*N*| values.

### 2.2 The propensity predicts the duration of the next REM bout

**Figure 4:**
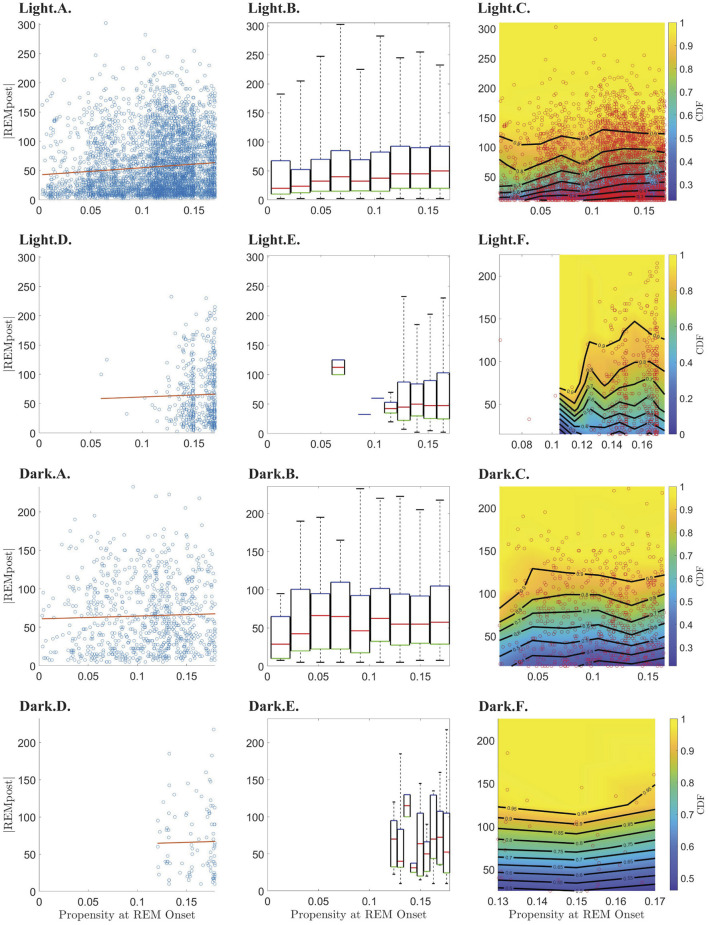
Relationship between REM propensity at REM onset and the duration (|*REMpost*|) of the subsequent REM bout. Rows 1–4 show the relationship between the REM propensity and |*REMpost*| for, respectively, from top to bottom, data before the peak in propensity during the light phase; data after the peak in propensity during the light phase; data before the peak in propensity during the dark phase; and data after the peak in propensity during the dark phase. Columns 1–3, from left to right, show, respectively, scatter plots (blue circles) of |*REMpost*| vs. REM propensity along with the line of best fit (red line) as calculated by linear regression; box-and-whisker plots for |*REMpost*| binned according to REM propensity; and CDFs (heat-color) and the corresponding contours (black lines) for |*REMpost*| binned according to REM propensity, using the same bins as the second column, with the scatter plot data (red circles) overlaid.

To test the hypothesis that, until it reaches its peak, the propensity drives an hourglass-like homeostatic pressure to enter REM sleep, we investigated the relationship between the propensity value at the onset of REMpost and REMpost duration, |*REMpost*|. We expected that if the propensity represents an hourglass-like homeostatic pressure to enter REM sleep, then a higher propensity at REM onset should lead to a longer |*REMpost*| (Park et al., 2021). We find that this relationship holds (Figure 4), but only during the light period (Supplementary Figure S3), and only for cycles with |*N*| less than the value of |*N*| at peak propensity (see Supplementary Figure S3 vs. Supplementary Figure S5).

Indeed, the top row of Figure 4 (panels Light.A.—Light.C.) shows that higher propensities are associated with longer |*REMpost*| during the light period. This positive correlation is seen in the raw data (Light.A.) and when propensity values are binned (Light.B.,C.). As shown in Table 1, there is a small but highly statistically significant positive correlation between propensities and |*REMpost*| for REM cycles whose |*N*| is smaller than the |*N*| at which propensity peaks. However, Table 1 and row 2 of Figure 4, (panels Light.D - Light.F), indicate that after the propensity peak, there is no significant correlation between |*REMpost*| and propensity at REM onset. Interestingly, rows 3–4 of Figure 4, (i.e., panels Dark.A.–Dark.F.), and Table 1 indicate that there is also no correlation between propensity and |*REMpost*| during the dark period.

**Table 1:** Correlation between REM propensity and features of REM sleep.

**Correlation between REM propensity and:**	**Light or dark**	**Before or after peak propensity**	**Pearson's corr. coefficient**	***P*-value**
REMpost	Light	Before	0.0977	3.5632·10^−10^
		After	n.s. (0.0205)	0.6560
	Dark	Before	n.s. (0.0345)	0.3267
		After	n.s. (0.0175)	0.8666
# of consecutive sequential cycles	Light	Both	0.0751	5.4818·10^−6^
		Before	0.0795	5.2222·10^−6^
	After	n.s. (0.0308)	0.5494
	Dark	Both	n.s. (0.0172)	0.6232
Before		n.s. (0.0269)	0.4661
After	n.s. (−0.1265)	0.2517
Probability of a sequential cycle	Light	Both	0.0740	7.5989·10^−6^
		Before	0.0776	8.6799·10^−6^
	After	n.s. (0.0405)	0.4316
	Dark	Both	n.s. (0.0226)	0.5176
Before		n.s. (0.0362)	0.3267
After	n.s. (−0.1265)	0.2517
# of consecutive sequential cycles in chains	Light	Both	n.s. (0.0592)	0.1234
		Before	n.s. (0.0659)	0.1040
	After	n.s. (−0.0257)	0.8350
	Dark	Both	n.s. (-0.0280)	0.8102
Before		n.s. (−0.0480)	0.6953
After	–	–

Non-significant correlation coefficients are indicated by n.s., followed by the corresponding coefficient in parentheses. Correlation coefficients that could not be computed due to lack of data are denoted by “–”.

### 2.3 The propensity predicts whether the next REM cycle is sequential

**Figure 5:**
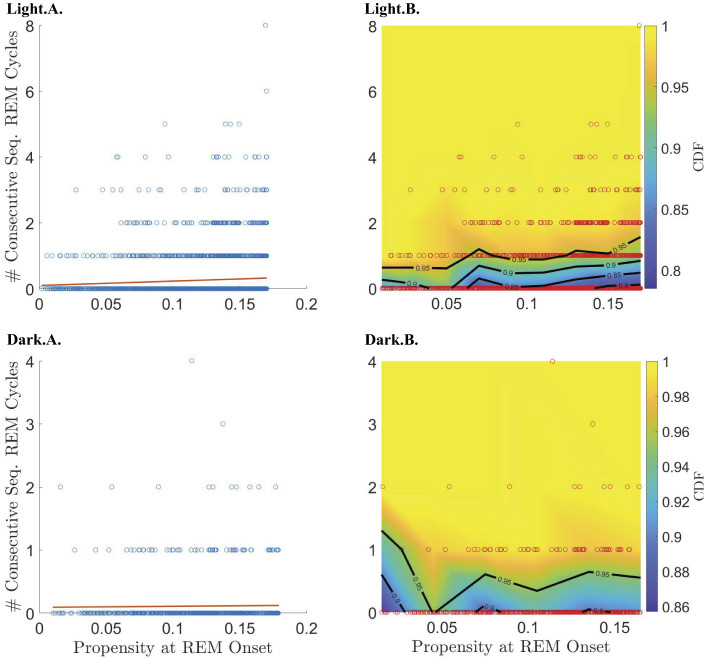
Relationship between REM propensity and the number of sequential cycles that follow. Rows 1 and 2 describe the relationship between the REM propensity at the end of a single cycle and the number of consecutive sequential REM cycles that follow, for the light and dark phases, respectively, for all REM cycles regardless of whether the corresponding REM propensity comes before or after the peak REM propensity. Columns 1–2, from left to right, show, respectively, scatter plots (blue circles) of the number of sequential REMs (if any) that follow a single cycle vs. REM propensity along with the line of best fit (red line) as calculated by linear regression; and CDFs (heat-color) and the corresponding contours (black lines) for the number of consecutive sequential REMs (if any) that follow, binned according to REM propensity, with the scatter plot data (red circles) overlaid.

To further investigate the hypothesis that the propensity represents a homeostatic pressure to enter REM sleep, we explored the relationship between the propensity at the end of a single REM cycle and the number |*S*| of consecutive sequential REM cycles that follow, (i.e., the length of a “chain” of sequential REMs; |*S*| = 0 means that the single REM cycle was followed by another single REM cycle). We found that the propensity has a small but highly significant correlation with |*S*| for propensities less than the peak propensity value (Table 1; Supplementary Figures S3, S5) and only during the light period (Figure 5; Table 1). This lends further support to the hypothesis that propensity represents a homeostatic pressure to enter REM sleep only for inter-REM intervals preceding the peak in propensity.

**Figure 6:**
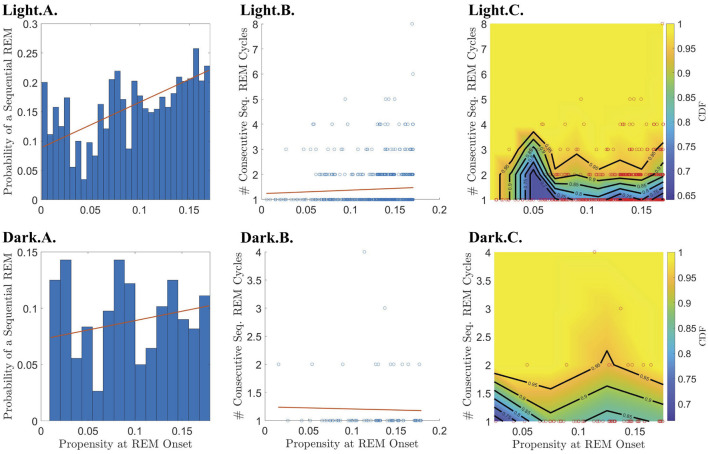
Relationship between REM propensity and the length of subsequent sequential chains vs. the probability that a sequential chain occurs. Rows 1 and 2 describe the relationship between the REM propensity at the end of a single cycle and the chain of consecutive sequential REM cycles that follows (if present), for the light and dark phases, respectively, for all REM cycles regardless of whether the corresponding REM propensity comes before or after the peak REM propensity. The leftmost column displays a bar chart of the probability that a sequential REM bout follows a single REM bout across bins of REM propensities at the onset of REM sleep at the end of the single cycle. The red best-fit line shows a positive (and statistically significant) slope during the light phase indicating that the probability of having a sequential REM increases with REM propensity. Columns 2–3, from left to right, show, respectively, scatter plots (blue circles) of REM propensity vs. the number of sequential REMs that follow a single REM cycle given that at least one sequential REM cycle follows along with the line of best fit (red line) as calculated by linear regression; and CDFs (heat-color) and the corresponding contours (black lines) for the number of sequential REM cycles binned according to REM propensity, with the scatter plot data (red circles) overlaid.

To break down the relationship between REM propensity and |*S*|, we separately investigated the relationship between propensity and (1) whether there is a subsequent sequential REM cycle at all, and (2) the number of sequential REM cycles assuming that there is at least one subsequent sequential REM cycle. We find that there is a statistically significant positive correlation between the propensity at REM onset and the probability of a subsequent sequential REM cycle (Table 1 and the Light.A. panel of Figure 6) during the light period for |N| before the REM propensity peaks (Table 1; Supplementary Figures S4, S6). In fact, the probability of a subsequent sequential REM cycle more than doubles as propensity increases to its peak value across all REM cycles occurring during the light phase. However, assuming that there is at least one subsequent sequential REM cycle during the light phase, the positive correlation between propensity and |*S*| (Light.B. and Light.C. panels of Figure 5), while trending, is not statistically significant (Table 1). Correlations between REM propensity and |*S*| during the dark period are not significant (Figure 5; Supplementary Figure S4), nor are correlations between REM propensity and |*S*| after the propensity reaches its peak (see Supplementary Figure S4 vs. Supplementary Figure S6).

To investigate why REM propensity correlates with the presence of a subsequent sequential REM, we check whether sequential REM cycles contain proportionally more REM sleep than single REM cycles. We find that, on average, during the light phase, about 11.73% of sleep occurring during single cycles is REM sleep, whereas almost three times that much–31.57%–of sleep occurring during sequential cycles is REM sleep (*p* = 0.0029, Welch's *t*-test, Table 2). An even higher percentage–32.32% on average–of sleep during chains of sequential cycles is REM (Table 2). Similarly, during the dark phase, the percentage of sleep that is REM is roughly 3 times larger during sequential REM cycles than during single REM cycles (Table 2). Thus, proportionally more REM sleep occurs during sequential cycles and chains of sequential cycles compared to single cycles.

**Table 2:** The proportion of sleep spent in REM for single cycles, sequential cycles, and chains of sequential cycles.

**Light or dark**	**Proportion of sleep that is REM**			***P*-value for single vs. sequential**	***P*-value for single vs. sequential chain**
	**Single cycles**	**Sequential cycles**	**Sequential chains**		
Light	0.1173	0.3157	0.3232	0.0029	0.0085
Dark	0.1190	0.4088	0.3966	5.8008·10^−6^	1.9405·10^−5^

There is a statistically significant difference in the proportion of sleep that is REM for single cycles vs. sequential cycles, and for single cycles vs. chains of sequential cycles. Statistical significance was determined using the two-tailed version of Welch's *t*-test.

## 3 Discussion

We have developed a new measure for the propensity to enter REM sleep (Equation 1). Our propensity measure depends only on the accumulation of time spent in NREM sleep up to the present, and therefore has the potential to be predictive, in contrast to other previous measures of REM sleep need. In addition, unlike previously proposed REM sleep propensity measures, our proposed REM propensity measure increases, attains a local maximum, and then decreases to zero as |*N*| increases (Figure 3). We also find that the propensity reflects the presence of sequential REMs by e.g., initially decreasing after short NREM accumulation.

Like Park et al. (2021), we find that our REM propensity at REM onset is correlated with the duration |*REMpost*| of the subsequent REM bout (Figure 4; Table 1). However, we find that the correlation is only statistically significant during the light phase, and only before the REM propensity peaks (Figure 4; Table 1).

Furthermore, the REM propensity is correlated with the number |*S*| of sequential REM cycles that follow a single REM episode (Figure 5; Table 1). However, upon closer examination, we find that this relationship reflects a correlation between propensity and the probability of a subsequent sequential REM (Figure 6; Table 1). Like the correlation between propensity and |*REMpost*|, the correlations between propensity and |*S*| and between propensity and the probability of a subsequent sequential REM cycle are only statistically significant during the light phase, and only over ranges where the REM propensity is increasing (Supplementary Figures S3–S6, as well as Table 1). Providing a potential reason as to why the REM propensity correlates with the presence of a subsequent sequential REM cycle, we find that a higher proportion of sleep is REM during sequential REM cycles and chains of sequential REM cycles than during single REM cycles.

Our analyses collectively highlight the properties and the usefulness of our proposed REM propensity measure. Our results also provide evidence for the hypothesis that a homeostatic REM pressure accumulates during NREM sleep, driving both longer durations of the subsequent REM episode and the presence of sequential REM cycles during the light phase. However, our results suggest that after sufficient NREM accumulation, REM pressure ceases to increase with amount of NREM sleep. The absence of significant correlations in the dark phase data suggest that the influence of the duration of NREM sleep on REM pressure is weaker in the dark phase.

### 3.1 Unique properties of the proposed REM propensity measure

Our proposed REM propensity measure mathematically represents the probability of entering REM sleep in the near future. To our knowledge we are the first group to apply this measure to data describing REM sleep. Similar formulas to our proposed propensity, however, have been used in the study of survival analysis. Specifically, the propensity definition we use is similar to the definition of the hazard function (see e.g., Clark et al., 2003 for a discussion of the hazard function in the study of survival analysis). However, the propensity definition we use differs in key ways from the hazard function. Most significantly, while the propensity we use exactly describes the probability of entering REM sleep in the near future, the hazard function can be used to approximate that probability (see Supplementary material S4 for more details).

A key advantage of the proposed REM propensity measure is its predictive power to describe how likely it is that REM sleep will occur as well as features of the following REM bout. This is a significant departure from previous measures of REM propensity which tended to be descriptive. Given the proposed REM propensity's predictive power, it could be used in a probabilistic model of ultradian sleep-state switching. Future work implementing real-time assessment of REM propensity may be able to gauge the probability that a transition to REM sleep occurs at a specific time point.

Our proposed REM propensity has several unique features. It is non-monotonic, has a local maximum, and eventually decays to zero. For smaller values of |*REMpre*|, the REM propensity measure may also exhibit a local minimum reflecting the presence of sequential REM cycles. Additionally, the decreases exhibited by our REM propensity measure differ from previously proposed measures of homeostatic REM sleep drive, such as the CDF of |*N*|, which increases monotonically by definition. Since the duration of NREM sleep since the last REM bout has been proposed to drive an hourglass-like process to enter REM sleep (Benington and Heller, 1994; Vivaldi et al., 1994, 2005; Franken, 2002; Park et al., 2021), the propensity to enter REM sleep based on |*N*| should increase as |*N*| increases. By contrast, our REM propensity measure decreases with sufficiently long time spent in NREM sleep, suggesting that |*N*| may drive an hourglass-like homeostatic REM pressure but only for a limited range of |*N*| durations. The REM propensity decaying to 0 as |*N*| becomes sufficiently large suggests that, for longer |*N*| durations, other factors affect the transition to REM more than the NREM duration does. In particular, other physiological or neurological factors, such as circadian rhythms, might begin to play a more dominant role in determining sleep cycle transitions. Moreover, inter-REM intervals with longer |*N*| durations are more likely to include more microarousals or longer wake episodes compared to intervals with shorter |*N*| durations, which could also play a role.

### 3.2 Homeostatic REM pressure accumulates during NREM sleep only during the light phase and while the propensity is increasing

Our analysis suggests that NREM sleep since the last REM bout does drive an hourglass-like REM pressure, but only during the light phase and only before REM propensity peaks. Specifically, if a higher REM propensity indicates a greater build-up of homeostatic REM pressure during NREM sleep, then a longer REM bout should follow, so as to dispel the elevated REM pressure. We find that to be the case, specifically during the light phase and before propensity reaches its peak. Interestingly, we also find that specifically during the light phase and before peak propensity, this REM propensity measure is also correlated with the presence of a subsequent sequential REM cycle. Since after the propensity begins to decay it is no longer predictive of the duration of the subsequent REM bout nor of the presence of a subsequent sequential REMs, we surmise that after enough NREM sleep has passed, |*N*| ceases to predict REM homeostatic need, or that its effects may be obscured by other factors.

Interestingly, the proposed REM propensity measure is not predictive of features of the subsequent REM bout during the dark period. This suggests that during the dark period, other factors contribute to driving transitions into REM sleep. We suspect that since mice are active during the dark period, confounding behavioral factors such as locomotion or eating may affect the need to enter REM sleep as much as the amount of |*N*| that has passed, explaining the lack of correlation between propensity and |*REMpost*|. Similarly, confounding factors may play a greater role when |*N*| is longer.

Additionally, the correlations between the REM propensity measure and features of the subsequent REM bout, including its duration and whether it is a sequential REM bout, while statistically significant, are low. This suggests that even when the REM propensity measure is predictive, there are likely other factors that also influence transitions to REM sleep. Such confounding factors could include ambient temperature (Amici et al., 1994), time of day (Merica and Gaillard, 1991), or circadian phase (Khalsa et al., 2002). Wake episodes and microarousals interspersed with sleep could also disrupt the drive to REM sleep. In particular, wake episodes have been linked with delayed REM transitions on the ultradian timescale (Park et al., 2021). However, Park et al. (2021) found that the presence of wake had no effect on the correlation between the CDF of |*N*| and the duration |*REMpost*| of the subsequent REM bout.

While our REM propensity measure supports the hypothesis of a short-term REM homeostatic drive, the absence of identified processes underlying REM homeostasis has prompted the proposal of alternative mechanisms generating NREM-REM alternations. For example, motivated by the strong positive correlation between the duration of a REM bout and the duration of the following inter-REM interval (Benington and Heller, 1994; Vivaldi et al., 1994), Le Bon (2021) proposed an asymmetrical hypothesis in which REM episodes are followed by a post-REM refractory period that limits when the next REM episode can occur. Alternatively, alternations in EEG power in the delta and theta frequency bands, that are associated with periods of NREM sleep and REM sleep or wake, respectively, have been shown to display features of self-organized criticality (Wang et al., 2019; Lombardi et al., 2020; Huo et al., 2024), a dynamical state that has been proposed to explain multiple features of brain activity dynamics (see Plenz et al., 2021 for a review), including during sleep (Comte et al., 2006; Priesemann et al., 2013; Scarpetta et al., 2023). A primary feature suggesting that cortical dynamics are positioned in a critical regime across wake and sleep states is that the durations of high theta power episodes (presumably wake or REM sleep bouts) show a power-law distribution and the durations of high delta power episodes (presumably NREM sleep bouts) show a Weibull (exponential) distribution (Wang et al., 2019; Lombardi et al., 2020; Huo et al., 2024). Interestingly, these authors find that obtaining these scaling behaviors for duration distributions depends on the durations of consecutive high delta and high theta episodes being anti-correlated, namely that long delta episodes are followed by short theta episodes. While this contrasts with our finding of higher REM propensity based on longer time in NREM sleep being correlated with longer REM bouts, the multiple differences in the data and analysis methods between those studies and ours make a direct comparison inappropriate. Discriminating among these competing theories for mechanisms governing NREM-REM alternation will require continued experimental study to identify brain regions and processes responsible for the short-term temporal architecture of sleep.

### 3.3 Higher REM propensity correlates with sequential REMs

The observation that a higher propensity correlates with the occurence of sequential REM bouts (in the light phase and before peak propensity) suggests that chains of sequential REM bouts may help to dispel REM pressure. There are several findings in the literature that support this hypothesis. Namely, in rats, sleep deprivation leads to an increase in the number of sequential REM cycles, but not in the number of single REM cycles (Zamboni et al., 1999). Similarly, REM sleep deprivation due to cold exposure leads to a rebound in REM sleep manifested by an increased number of sequential REM cycles, but not in the number of single REM bouts (Amici et al., 1994). Indeed, increased sequential REM bouts would preferentially help to dispel REM pressure if more REM sleep occurs during intervals with sequential REM cycles compared to intervals with single REM cycles. Our results support this notion, since in the mouse sleep data from Park et al. (2021), we find that 3 times more of sleep during sequential cycles and chains of sequential cycles is REM compared to in single cycles, and that this difference is statistically significant. Thus, sequential REM cycles present a much more efficient way to get more REM sleep compared to single cycles, supporting the notion that chains of sequential REMs help dispel REM pressure.

### 3.4 Limitations

The use of GMMs to describe the distribution of |*N*| may affect the accuracy of the REM propensity measure. For example, computing the propensity based on discrete bins of |*REMpre*| introduces error by potentially ignoring finer time-scale dependencies on |*REMpre*| durations. This error is likely small, but it has the potential to be more significant for smaller REMpre bins which contain more |*N*| data than other bins and whose |*REMpre*| values correspond to regions where the GMM fits change most rapidly (Supplementary Figure S2).

Further, the GMM fit for REM cycles occurring during the light period when |*REMpre*| ∈[60, 90) s, while qualitatively reproducing the distribution of |*N*|, fails the Kolmogorov-Smirnov test. Thus, for the GMM for that particular bin, the REM cycles that comprise the data are not clearly divided into sequential REM and single REM episodes. We suspect that the GMM fit fails the Kolmogorov-Smirnov test for that particular bin due to an asymmetric peak in the PDF of |*N*| (Supplementary Figure S1), which we suspect to be a random fluctuation in the data that would vanish with more data for longer |*N*|s. Along similar lines, we have little data on REM cycles occurring during the dark phase when |*REMpre*| exceeds 180 s. As a result, the GMM fit for that bin might not well-represent the true distribution of |*N*|, even though the fit does pass the Kolmogorov-Smirnov test. Nevertheless, since there are so few data points for this bin, the possibility that the GMM fit might not well-represent the true distribution of |*N*| likely has no significant impact on our conclusions.

Also noteworthy is that *k*_*l*_ is larger for bins corresponding to longer REMpre durations, suggesting that when |*REMpre*| is long, sequential cycles are unlikely. Thus, the parameters μ_*s*_ and σ_*s*_ corresponding to the “short” distribution comprising the GMM for those bins have little impact on the actual shape of the GMM. We suspect that if we had more data for those bins, that the values of μ_*s*_ and σ_*s*_ would lie roughly on a line extended from the values of μ_*s*_ and σ_*s*_ for bins with smaller |*REMpre*| values (Supplementary Figure S2).

### 3.5 Conclusions

We have defined a new, predictive REM propensity measure that depends only on the time spent in NREM sleep between REM bouts. We have demonstrated that this measure reflects the likelihood of entering REM sleep in the near future and predicts features of the next REM bout. Namely, a higher REM propensity measure predicts that the next REM bout will be longer and is more likely to belong to a sequential REM cycle. This REM propensity measure also suggests that, during the light phase, the amount of NREM sleep drives an hourglass-like REM pressure only up to a certain time, namely for NREM sleep amounts corresponding to the increasing phase of REM propensity.

Application of this REM propensity measure to additional data sets will further test its accuracy and usefulness. For example, the measure has potential to quantify effects of REM sleep deprivation on sleep architecture, where the amount of NREM sleep corresponding to the peak propensity is likely to be affected. Furthermore, applying the measure to sleep data from different mammalian species can test the overall prediction that REM sleep homeostasis depends primarily on NREM sleep duration.

## 4 Materials and methods

### 4.1 Data processing

All data processing and analysis was conducted with MATLAB. For each scored epoch in each of the 125 recordings, we labeled whether the mouse's environment was light or dark according to experimental protocol and the time stamp of the recording. We also lumped the sleep scores corresponding to various stages of NREM sleep into one general NREM sleep category. From the resulting scored data, we identified all REM cycles. However, to make sure the REM cycles occurred while the mice were primarily sleeping, we discarded REM cycles that included extended wake periods, interpreting “extended” to mean lasting longer than 5 min. We separately analyzed light and dark period data, and we identified 4,686 REM cycles during the light period and 927 REM cycles during the dark period. Note that the difference in the number of REM cycles between our model and between Park et al. (2021) occurs primarily because we exclude REM cycles with extended wake periods, whereas Park et al. (2021) does not. Also differing from Park et al. (2021), we interpreted microarousals as wake and not part of |*N*| when computing the inter-REM interval duration.

### 4.2 Fitting the data to a GMM

After dividing the data into the aforementioned REMpre bins, we fit a Gaussian mixture model (GMM) to the distribution of log|*N*| for each |*REMpre*| bin for the light and dark data separately by using an expectation-maximization algorithm. Also since Weibull distributions are a common choice for modeling the rate of state transitions or failures in survival analysis (Latimer, 2013), we tried to fit a 5-parameter Weibull mixture model to log|*N*| for each |*REMpre*| bin using an expectation-maximization algorithm (Elmahdy and Aboutahoun, 2013). However, using corrected Kolmogorov-Smirnov tests for the GMM fits (Park et al., 2021) and for the Weibull mixture model fits (Parsons and Wirsching, 1982), we found that the GMMs fit the data better compared to the Weibull mixture models (data not shown). As a result, we only report analysis of the data using GMMs.

We used the expectation-maximization algorithm to compute 20 fits of the GMM to the distribution of log(|*N*|), selecting initial choices of parameters uniformly at random from a reasonable range. From this collection of fits, we retained the fit that yielded the lowest Kolmogorov-Smirnov statistic. Each time we applied the expectation-maximization algorithm, we iterated the expectation and maximization steps repeatedly until the squared Euclidean distance between GMM parameter vectors (*k*_*l*_, μ_*l*_, σ_*l*_, μ_*s*_, σ_*s*_) across two successive iterations of the expectation and maximization steps was less than 10^−10^, as a criterion for the convergence of the algorithm. We then evaluated the goodness of fit of the GMM model for each |*REMpre*| bin using a Kolmogorov-Smirnov test with a Lilliefors-like correction (see Park et al., 2021).

### 4.3 Validation of the proposed REM propensity measure

To validate the proposed REM propensity measure, we divided all REM cycle data for the light phase into a training set consisting of 80% of the REM cycles, and a test set consisting of the remaining 20% of the REM cycles. We computed a GMM for each REMpre bin of the training data set exactly as we did in Section 4.2 (finding that each GMM fit passed the Kolmogorov-Smirnov test), and from each GMM we computed the corresponding propensity measure. From the test data, we computed an empirical propensity measure for each REMpre bin, not from the GMM, but directly from the test data as follows:


$$\begin{array}{l} \text{Empirical Propensity}=\\ \frac{\text{\# of REM cycles transitioning from NREM to REM in the next 30 s}}{\text{\# of REM cycles that have yet to transition from NREM to REM}}. \end{array}$$

**Figure 7:**
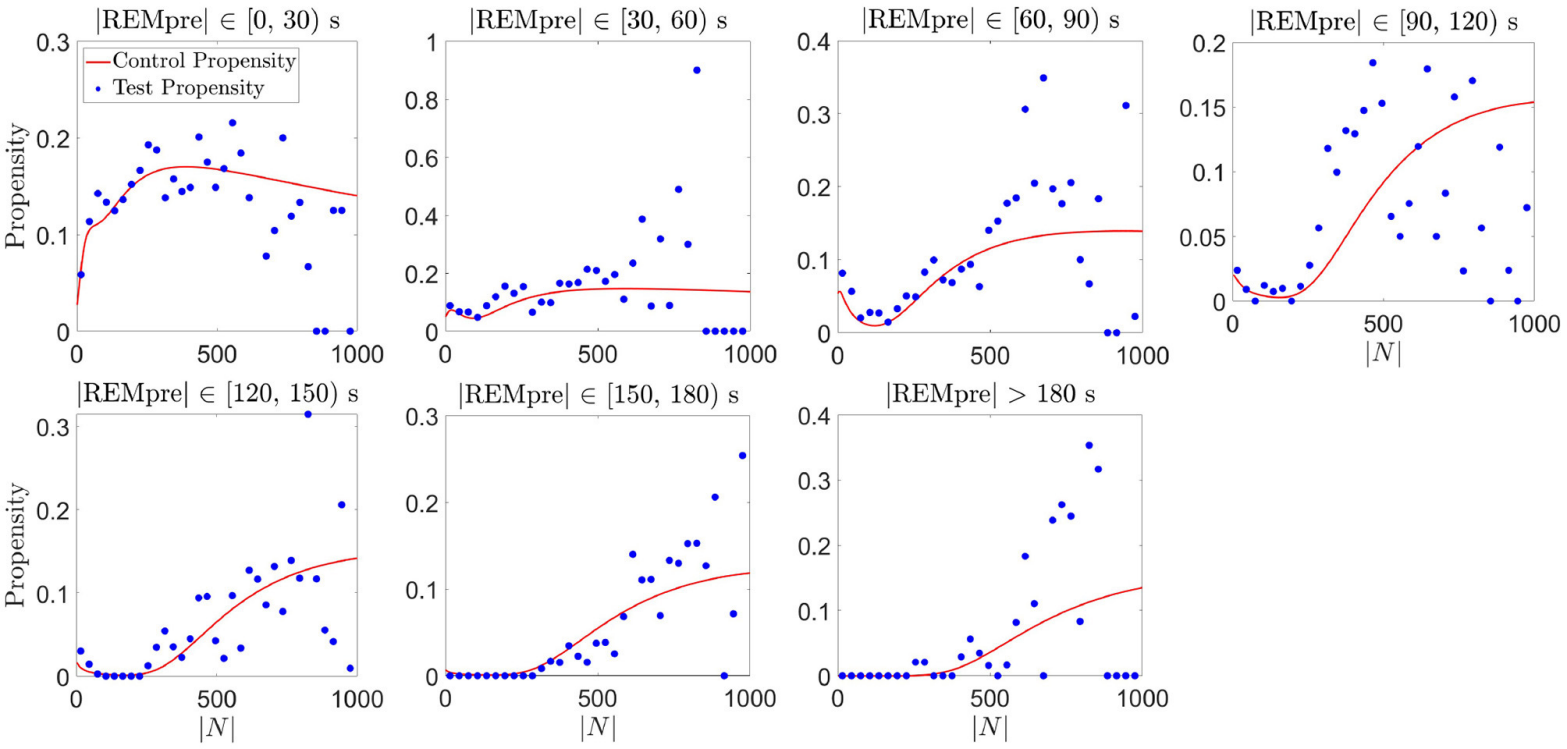
Validation of the proposed REM propensity measure. We consider REM propensity during the light phase. The red curve represents the propensity functions computed from the GMM fit of the CDF for |*N*| for each REMpre bin for a training data set consisting of 80% of all the REM cycles. The filled blue circles, on the other hand, show average empirical propensity values computed from a test data set consisting of the remaining 20% of the REM cycles (averages taken across 30 s bins of |*N*|).

This empirical propensity was computed at each second of the |*N*| interval. To reduce the noise present in the empirical propensity due to sparse data, particularly for large |*N*|, we computed the averages of the empirical propensities across 30 s bins of |*N*|. As shown in Figure 7, for each REMpre bin, average empirical propensities, while noisy, qualitatively matched the propensity measure based on the GMMs computed from the training data. Consequently, we expect that our GMM-based propensity measure can reasonably predict whether the C57BL/6J mice [the type of mice used in the Park et al. (2021) study], when housed in 12:12 h light:dark conditions, will transition from NREM to REM sleep in the next 30 s. Analogously, separating the data for the dark phase into a training set and a test set and computing GMM-based and empirical propensities yielded similar qualitative matching albeit with much noisier empirical propensities due to fewer data points (results not shown).

**Figure 8:**
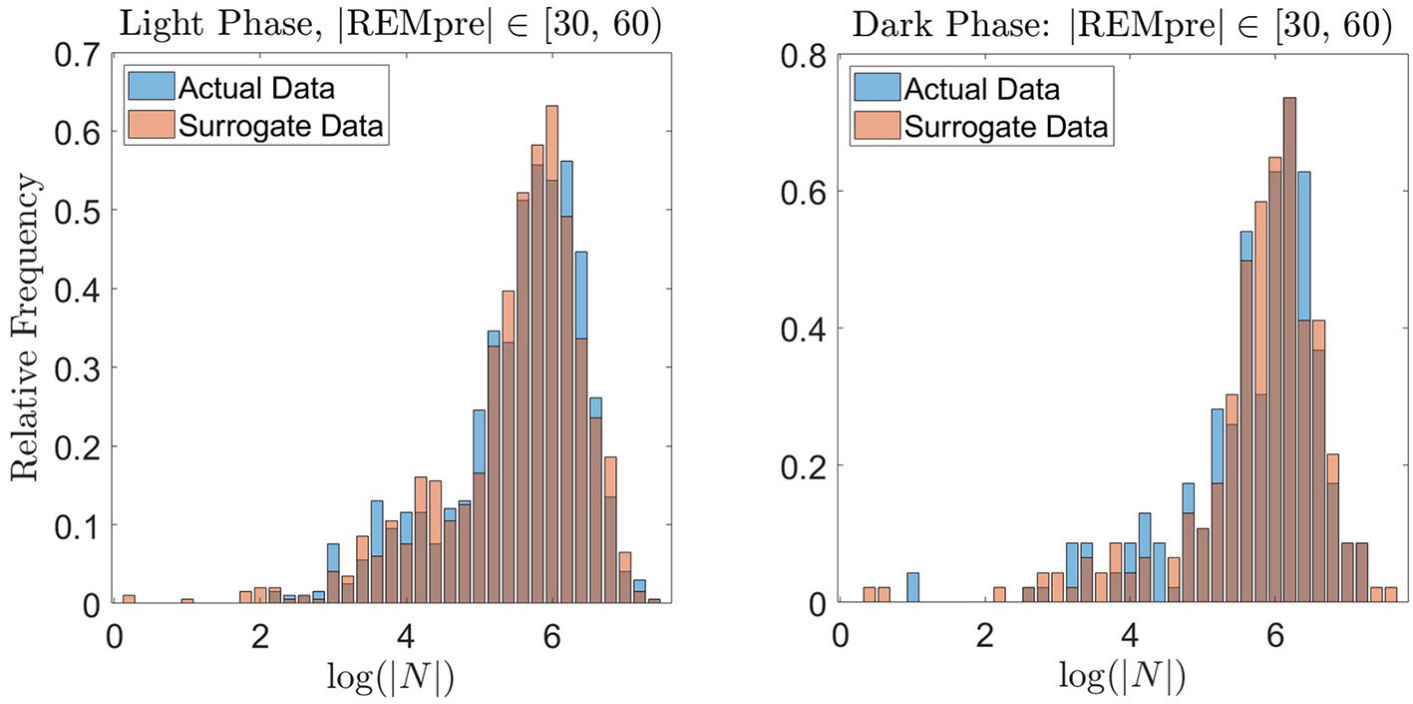
Validation of the proposed REM propensity measure via generation of surrogate data. We consider REM propensity during the light phase (left panel) and dark phase (right panel) separately, for |REMpre|∈[30, 60) s. The blue histograms (backgrounds) show the probability distribution of log(|*N*|) for the experimentally measured REM cycle data. The red histograms (foregrounds) show the probability distribution of log(|*N*|) calculated from a surrogate data set generated by the propensity functions.

To further validate the REM propensity measure, we compared the distribution of log(|*N*|) values from the REM cycle data to a distribution of log(|*N*|) values computed from an equally sized “surrogate” data set artificially generated by the propensity measure based on the GMM. For the surrogate data set, to generate a single value of |*N*|, we used the propensity to determine whether the transition to REM occurs in a 30 s interval. For example, if the REM propensity was 0.1 at time 1 s, then 10% of the time, |*N*|∈[1, 31). For the computation, we compared the value of the propensity at the beginning of each 30 s interval (starting at time 1 s) to the value of a uniform random number between 0 and 1. If the propensity exceeded the random number, then we considered the transition to REM to occur within the next 30 s, and selected |*N*| uniformly at random within that 30 s interval. If the propensity did not exceed the random number, then we considered the transition to REM to not have yet occurred, and repeated the preceding process for the subsequent 30 s interval choosing a new random number. We proceeded in this manner until an |*N*| was determined. As shown in Figure 8 for the light and dark data with |*REMpre*| ∈[30, 60) s, the resulting distribution of surrogate log(|*N*|) values qualitatively matches the distribution of log(|*N*|) exhibited in the experimentally measured REM cycle data. Similar results were obtained for the other REMpre bin data (results not shown).

### 4.4 Computing the relationship between the REM propensity measure and |*REMpost*|

As noted above, we separately analyzed light and dark phase data. For each REMpre bin, to determine whether |*N*| for each REM cycle came before or after the peak in propensity, we computed the largest |*N*| value at which the propensity for the corresponding |*REMpre*| bin has a local maximum. For the particular data sets that we were working with, this amounted to finding the value of |*N*| at the peak propensity for each bin. To do so, we computed the propensity for |*N*| = 1, 2, 3, …, 3, 000 s. Using MATLAB's built-in “islocalmax” function, we computed all values of |*N*| at which the propensity has a local maximum, and found the largest of the resulting values of |*N*|. Then, for each phase, we lumped REM cycles into two groups (across all |*REMpre*| bins) according to whether or not they came before or after the largest |*N*| at which there was a local maximum. For each of those two groups for each phase, we then found the Pearson's Correlation Coefficient between the REM propensity measure at REM onset and the duration (|*REMpost*|) of the subsequent REM cycle. We found the corresponding *p*-value using a two-tailed Student's *t*-test.

### 4.5 Computing the relationship between the REM propensity measure and consecutive sequential REMs that follow

For each |*REMpre*| bin for the light (dark) phase, we compute a cutoff-value of |*N*|, above which a REM cycle is labeled as a single REM cycle and below which the cycle is labeled as a sequential REM cycle. To compute the cutoff, we assume that the log(|*N*|) values come from one of two Gaussian distributions composing the GMM: *N*_*s*_, the portion of the GMM with a shorter mean |*N*|, or *N*_*l*_, the portion of the GMM with the longer mean |*N*|. Using this notation, GMM = *k*_*l*_*N*_*l*_+(1−*k*_*l*_)*N*_*s*_. We take the cutoff to be the value of |*N*| at which the probability that the REM cycle came from *N*_*s*_ equals the probability that the cycle came from *N*_*l*_. This cutoff is simply the value of |*N*| at the intersection between the *k*_*l*_*N*_*l*_ and (1−*k*_*l*_)*N*_*s*_ functions. However, for the light phase bin where |*REMpre*| >180 s, *N*_*l*_ and *N*_*s*_ have nearly the same mean, suggesting that all REM cycles in the bin may be of the same type. Also considering that the means of *N*_*s*_ and *N*_*l*_ for this bin are large and comparable to the means of *N*_*l*_ for the next shorter |*REMpre*| bins ∈[120, 150) s and [150, 180) s, we label all cycles for the bin with |*REMpre*| >180 s in the light phase as single. Similar issues arise for the dark phase, where *N*_*s*_ and *N*_*l*_ have similar means for the bins with |*REMpre*| >180 s and with |*REMpre*| ∈[150, 180) s. As a result, we approximate the cutoff value of |*N*| above which we labeled a cycle as single with the cutoff from a nearby bin. In particular, we set the cutoff value of |*N*| for the bin with |*REMpre*| >180 s to the cutoff for the bin with |*REMpre*| ∈[150, 180) s; and to set the cutoff value of |*N*| for the bin with |*REMpre*| [90, 120) s to the cutoff for the bin with |*REMpre*| ∈[120, 150) s.

For each single REM cycle in the light (dark) phase, we investigate three quantities: (1) we count the number of consecutive sequential REM bouts that follow, (2) we count the number of sequential REM bouts that follow assuming that there is at least one sequential REM that follows; and (3) we track whether a sequential REM cycle follows at all. We then compute the Pearson's Correlation Coefficient between the REM propensity measure at REM onset and each of those three quantities, and the corresponding *p*-value using a two-tailed Student's *t*-test. For the third quantity, we also compute a logistic regression between the REM propensity measure at REM onset and the presence of a subsequent sequential REM cycle (denoted by 1 if a sequential REM cycle follows and 0 if not). The logistic regression yielded qualitatively similar results to a linear regression, so we focused on the linear regression because we can interpret it using Pearson's correlation coefficient and report its statistical significance in the same manner we report statistical significance of other relationships. We repeat this analysis after lumping all single REM bouts within the light and dark periods, respectively.

### 4.6 Computing differences in the proportion of sleep spent in REM during sequential vs. single cycles

For each REM cycle in the light or dark phase, respectively, we computed the ratio of REM sleep duration to total sleep duration. We then computed the average ratios across single cycles, sequential cycles, and chains of sequential cycles. We applied Welch's *t*-test to determine whether the average ratio of REM sleep duration to total sleep duration differs between single and sequential REM cycles. We use a *p*-value of 0.05 as the cutoff for statistical significance, as we do elsewhere throughout the manuscript.

## Data Availability

Publicly available datasets were analyzed in this study. This data can be found here: https://zenodo.org/records/5817119#.YdvvNC_kGTc and https://zenodo.org/records/5820559#.Ydvvcy_kGTc.

## References

[B1] Amici R. Zamboni G. Perez E. Jones C. A. Toni I. Culin F.. (1994). Pattern of desynchronized sleep during deprivation and recovery induced in the rat by changes in ambient temperature. J. Sleep Res. 3, 250–256. 10.1111/j.1365-2869.1994.tb00139.x 10607133

[B2] Barbato G. Wehr T. A. (1998). Homeostatic regulation of REM sleep in humans during extended sleep. Sleep 21, 267–276. 10.1093/sleep/21.3.267 9595605

[B3] Bassi A. Vivaldi E. A. Ocampo-Garcés A. (2009). The time course of the probability of transition into and out of REM sleep. Sleep 32, 655–669. 10.1093/sleep/32.5.655 19480233 PMC2675901

[B4] Benington J. H. Heller H. C. (1994). REM-sleep timing is controlled homeostatically by accumulation of REM-sleep propensity in non-REM sleep. Am. J. Physiol. 266, R1992–R2000. 10.1152/ajpregu.1994.266.6.R1992 8024056

[B5] Benington J. H. Woudenberg M. C. Heller H. C. (1994). REM-sleep propensity accumulates during 2-h REM-sleep deprivation in the rest period in rats. Neurosci. Lett. 180, 76–80. 10.1016/0304-3940(94)90917-2 7877767

[B6] Cajochen C. Reichert C. F. Münch M. Gabel V. Stefani O. Chellappa S. L.. (2023). Ultradian sleep cycles: Frequency, duration, and associations with individual and environmental factors—A retrospective study. Sleep Health 10, S52–S62. 10.1016/j.sleh.2023.09.002 37914631

[B7] Chang A.-M. Aeschbach D. Duffy J. F. Czeisler C. A. (2015). Evening use of light-emitting eReaders negatively affects sleep, circadian timing, and next-morning alertness. Proc. Nat. Acad. Sci. U. S. A. 112, 1232–1237. 10.1073/pnas.1418490112 25535358 PMC4313820

[B8] Clark T. G. Bradburn M. J. Love S. B. Altman D. G. (2003). Survival analysis part I: basic concepts and first analyses. Br. J. Cancer 89, 232–238. 10.1038/sj.bjc.6601118 12865907 PMC2394262

[B9] Comte J. C. Ravassard P. Salin P. A. (2006). Sleep dynamics: a self-organized critical system. Phys. Rev. E 73:056127. 10.1103/PhysRevE.73.056127 16803018

[B10] Dijk D.-J. Groeger J. A. Stanley N. Deacon S. (2010). Age-related reduction in daytime sleep propensity and nocturnal slow wave sleep. Sleep 33, 211–223. 10.1093/sleep/33.2.211 20175405 PMC2817908

[B11] Elmahdy E. E. Aboutahoun A. W. (2013). A new approach for parameter estimation of finite Weibull mixture distributions for reliability modeling. Appl. Math. Model. 37, 1800–1810. 10.1016/j.apm.2012.04.023

[B12] Franken P. (2002). Long-term vs. short-term processes regulating REM sleep. J. Sleep Res. 11, 17–28. 10.1046/j.1365-2869.2002.00275.x 11869422

[B13] Gregory G. G. Cabeza R. (2002). A two-state stochastic model of REM sleep architecture in the rat. J. Neurophysiol. 88, 2589–2597. 10.1152/jn.00861.2001 12424296

[B14] Heller C. (2021). The Regulation of Sleep. Oxford University Press. 10.1093/acrefore/9780190264086.013.30

[B15] Huo C. Lombardi F. Blanco-Centurion C. Shiromani P. J. Ivanov P. C. (2024). Role of the LC arousal promoting neurons in maintaining brain criticality across the sleep-wake cycle. J. Neurosci. e1939232024. 10.1523/JNEUROSCI.1939-23.2024 38951035 PMC11358608

[B16] Khalsa S. B. S. Conroy D. A. Duffy J. F. Czeisler C. A. Dij,K D.-J. (2002). Sleep-and circadian-dependent modulation of REM density. J. Sleep Res. 11, 53–59. 10.1046/j.1365-2869.2002.00276.x 11869427

[B17] Kripke D. Reite M. Pegram G. Stephens L. Lewis O. (1968). Nocturnal sleep in rhesus monkeys. Electroencephalogr. Clin. Neurophysiol. 24, 581–586. 10.1016/0013-4694(68)90047-3 4172742

[B18] Latimer N. R. (2013). Survival analysis for economic evaluations alongside clinical trials—extrapolation with patient-level data: inconsistencies, limitations, and a practical guide. Med. Decis. Making 33, 743–754. 10.1177/0272989X12472398 23341049

[B19] Le Bon O. (2021). An asymmetrical hypothesis for the NREM-REM sleep alternation—what is the NREM-REM cycle? Front. Neurosci. 15:627193. 10.3389/fnins.2021.627193 33897348 PMC8060555

[B20] Lombardi F. Gómez-Extremera M. Bernaola-Galván P. Vetrivelan R. Saper C. B. Scammell T. E.. (2020). Critical dynamics and coupling in bursts of cortical rhythms indicate non-homeostatic mechanism for sleep-stage transitions and dual role of VLPO neurons in both sleep and wake. J. Neurosci. 40, 171–190. 10.1523/JNEUROSCI.1278-19.2019 31694962 PMC6939478

[B21] Merica H. Gaillard J.-M. (1991). A study of the interrupted REM episode. Physiol. Behav. 50, 1153–1159. 10.1016/0031-9384(91)90576-A 1798770

[B22] Nielsen T. A. Paquette T. Solomonova E. Lara-Carrasco J. Popova A. Levrier K. (2010). REM sleep characteristics of nightmare sufferers before and after REM sleep deprivation. Sleep Med. 11, 172–179. 10.1016/j.sleep.2008.12.018 20005773

[B23] Park S.-H. Baik J. Hong J. Antila H. Kurland B. Chung S.. (2021). A probabilistic model for the ultradian timing of REM sleep in mice. PLoS Comput. Biol. 17, e1009316. 10.1371/journal.pcbi.1009316 34432801 PMC8423363

[B24] Parsons F. Wirsching P. (1982). A Kolmogorov-Smirnov goodness-of-fit test for the two-parameter weibull distribution when the parameters are estimated from the data. Microelectron. Reliab. 22, 163–167. 10.1016/0026-2714(82)90174-338469434

[B25] Plenz D. Ribeiro T. L. Miller S. R. Kells P. A. Vakili A. Capek E. L. (2021). Self-organized criticality in the brain. Front. Phys. 9:639389. 10.3389/fphy.2021.639389

[B26] Priesemann V. Valderrama M. Wibral M. Le Van Quyen M. (2013). Neuronal avalanches differ from wakefulness to deep sleep—evidence from intracranial depth recordings in humans. PLoS Comput. Biol. 9, 1–14. 10.1186/1471-2202-14-S1-P237 23555220 PMC3605058

[B27] Scarpetta S. Morisi N. Mutti C. Azzi N. Trippi I. Ciliento R.. (2023). Criticality of neuronal avalanches in human sleep and their relationship with sleep macro- and micro-architecture. iScience 26:107840. 10.1016/j.isci.2023.107840 37766992 PMC10520337

[B28] Ursin R. (1970). Sleep stage relations within the sleep cycles of the cat. Brain Res. 20, 91–97. 10.1016/0006-8993(70)90157-5 4315521

[B29] Vivaldi E. A. Ocampo A. Wyneken U. Roncagliolo M. Zapata A. (1994). Short-term homeostasis of active sleep and the architecture of sleep in the rat. J. Neurophysiol. 72, 1745–1755. 10.1152/jn.1994.72.4.1745 7823099

[B30] Vivaldi E. A. Ocampo-Garcés A. Villegas R. (2005). Short-term homeostasis of REM sleep throughout a 12: 12 light: dark schedule in the rat. Sleep 28, 931–943. 10.1093/sleep/28.8.931 16218076

[B31] Wang J. W. J. L. Lombardi F. Zhang X. Anaclet C. Ivanov P. C. (2019). Non-equilibrium critical dynamics of bursts in θ and δ rhythms as fundamental characteristic of sleep and wake micro-architecture. PLoS Comput. Biol. 15, 1–35. 10.1371/journal.pcbi.1007268 31725712 PMC6855414

[B32] Zamboni G. Perez E. Amici R. Jones C. Parmeggiani P. (1999). Control of REM sleep: an aspect of the regulation of physiological homeostasis. Arch. Ital. Biol. 137, 249–262. 10443317

