# Supplementary material for: A predictive propensity measure to enter REM sleep
Source: Front Neurosci. 2024 Aug 30;18:1431407. doi: 10.3389/fnins.2024.1431407 (PMC11392850; doi:10.3389/fnins.2024.1431407)
Supplement: Supplementary file 1 [file Data_Sheet_1.pdf]

Supplementary Material

S1. PROOF THAT THE PROPOSED REM PROPENSITY MEASURE DESCRIBES THE PROBABILITY OF ENTERING REM SLEEP IN THE "NEAR FUTURE"

We assume that REM propensity is driven by the accumulation of a biological substrate and we suppose that the accumulated value of this substrate at a point in time is x. Then, the probability of a state transition occurring within the next Δx units of accumulation is the probability that the value of x at the state transition, X_τ, is between x and $x + \Delta x$, i.e that $X_\tau \in (x, x + \Delta x]$, given that the state transition has not yet happened, i.e that $X_\tau > x$. Then, from Bayes' law, the desired probability is

$$\begin{aligned}\mathbb{P}(\text{entering REM sleep in the next } \Delta x \text{ unit change}) &= \mathbb{P}\left(X_\tau \in (x, x+\Delta x] | X_\tau > x\right) \\ &= \frac{\mathbb{P}\left(X_\tau \in (x, x+\Delta x] \text{ and } X_\tau > x\right)}{\mathbb{P}(X_\tau > x)}.\end{aligned}$$

However, if $X_\tau \in (x, x + \Delta x]$, it is guaranteed that $X_\tau > x$. Consequently, $\mathbb{P}\left(X_\tau \in (x, x+\Delta x] \text{ and } X_\tau > x\right) = \mathbb{P}\left(X_\tau \in (x, x+\Delta x]\right)$, and so

$$\mathbb{P}(\text{entering REM sleep in the next } \Delta x \text{ unit change}) = \frac{\mathbb{P}\left(X_\tau \in (x, x+\Delta x]\right)}{\mathbb{P}(X_\tau > x)}.$$

We can rewrite the numerator and denominator of the preceding expression using CDFs. In particular, using basic properties of the CDF, we can rewrite the denominator as

$$\begin{aligned}\mathbb{P}(X_\tau > x) &= 1 - \mathbb{P}(X_\tau \leq x) \\ &= 1 - CDF(x).\end{aligned}$$

Similarly, we can rewrite the numerator as

$$\begin{aligned}\mathbb{P}\left(X_\tau \in (x, x+\Delta x]\right) &= \mathbb{P}(X_\tau \leq x + \Delta x) - \mathbb{P}(X_\tau \leq x) \\ &= CDF(x + \Delta x) - CDF(x).\end{aligned}$$

Thus, the desired probability is

$$\begin{aligned}\mathbb{P}(\text{entering REM sleep in the next } \Delta x \text{ unit change}) &= \frac{CDF(x+\Delta x) - CDF(x)}{1 - CDF(x)} \\ &= p_{\Delta x}(x).\end{aligned}$$

We take this probability $p_{\Delta x}(x)$ to be our proposed REM propensity measure.

S2. CONDITIONS FOR $p_{\Delta x}(x)$ TO REPRESENT AN HOURGLASS PROCESS

It has been hypothesized that REM sleep pressure represents an hourglass process that increases in the absence of REM sleep and decreases during REM sleep. Based on this hypothesis, if our

propensity measure $p_{\Delta x}(x)$ reflects REM pressure, then it should increase monotonically. In the following subsections, we derive conditions for $p_{\Delta x}(x)$ to increase monotonically.

S2.1. Condition for $\boldsymbol{p_{\Delta x}(x)}$ to be strictly increasing

Assuming the CDF of the accumulating quantity underlying REM propensity is differentiable, then the measure $p_{\Delta x}(x)$ is strictly increasing if and only if its derivative is positive. Below, we explicitly compute the derivative of $p_{\Delta x}(x)$ and simplify to find a condition equivalent to $p_{\Delta x}(x)$ being strictly increasing:

$$
\begin{aligned}
p'_{\Delta x}(x) &= \frac{(CDF'(x+\Delta x) - CDF'(x))(1 - CDF(x)) + (CDF(x+\Delta x) - CDF(x))CDF'(x)}{(1 - CDF(x))^2} \\
&= \frac{(CDF'(x+\Delta x) - CDF'(x)) - CDF'(x+\Delta x)CDF(x) + CDF'(x)CDF(x) + CDF(x+\Delta x)CDF'(x) - CDF(x)CDF'(x)}{(1 - CDF(x))^2} \\
&= \frac{(CDF'(x+\Delta x) - CDF'(x)) - CDF'(x+\Delta x)CDF(x) + CDF(x+\Delta x)CDF'(x)}{(1 - CDF(x))^2} \\
&= \frac{CDF'(x+\Delta x)(1 - CDF(x)) - CDF'(x)(1 - CDF(x+\Delta x))}{(1 - CDF(x))^2}
\end{aligned}
$$

which is positive if and only if its numerator is

$$0 < CDF'(x+\Delta x)(1 - CDF(x)) - CDF'(x)(1 - CDF(x+\Delta x)).$$

We rewrite the preceding inequality using the corresponding PDF:

$$0 < PDF(x+\Delta x)(1 - CDF(x)) - PDF(x)(1 - CDF(x+\Delta x)).$$

Dividing through by $PDF(x)$ and $1 - CDF(x)$ yields

$$0 < \frac{PDF(x+\Delta x)}{PDF(x)} - \frac{1 - CDF(x+\Delta x)}{1 - CDF(x)},$$

so

$$\frac{1 - CDF(x+\Delta x)}{1 - CDF(x)} < \frac{PDF(x+\Delta x)}{PDF(x)},$$

and equivalently

$$\frac{\int_{x+\Delta x}^{\infty} PDF(s)ds}{\int_{x}^{\infty} PDF(s)ds} < \frac{PDF(x+\Delta x)}{PDF(x)}.$$

To simplify the integrals, we now make the change of variable $u = s - \Delta x$ in the numerator of the left-hand side. Then, when $s = x + \Delta x$, we get that $u = x$, and the left-hand side of the preceding

expression

$$\frac{\int_{x+\Delta x}^{\infty} PDF(s)ds}{\int_{x}^{\infty} PDF(s)ds} = \frac{\int_{x}^{\infty} PDF(u+\Delta x)du}{\int_{x}^{\infty} PDF(s)ds} = \frac{\int_{x}^{\infty} PDF(s+\Delta x)ds}{\int_{x}^{\infty} PDF(s)ds}.$$

Thus, $p_{\Delta x}(x)$ is strictly increasing if and only if

$$\frac{\int_{x}^{\infty} PDF(s+\Delta x)ds}{\int_{x}^{\infty} PDF(s)ds} < \frac{PDF(x+\Delta x)}{PDF(x)}. \tag{S1}$$

S2.2. Sufficient condition for $\boldsymbol{p_{\Delta x}(x)}$ to be strictly increasing

To find a sufficient condition for $p_{\Delta x}(x)$ to be strictly increasing, we seek to find a scenario in which the equivalent condition (Eq. S1) holds. Specifically, we will argue that if $\frac{PDF(x+\Delta x)}{PDF(x)}$ is strictly decreasing in x, $p_{\Delta x}(x)$ is strictly increasing in x. To start, we define the right-hand side of Eq. S1 to be

$$R(x, \Delta x) = \frac{PDF(x+\Delta x)}{PDF(x)},$$

and seek to hold that Eq. S1 holds. To do so, we first work with the numerator of the left-hand-side of Eq. S1. Specifically, if $R(x, \Delta x)$ is strictly decreasing in x, then for $x < s$, we have $R(s, \Delta x) < R(x, \Delta x)$, implying that

$$\int_{x}^{\infty} PDF(s+\Delta x)ds = \int_{x}^{\infty} PDF(s)R(s, \Delta x)ds < \int_{x}^{\infty} PDF(s)R(x, \Delta x)ds,$$

noting that the PDF and R are always non-negative. Dividing the preceding inequality through by $\int_{x}^{\infty} PDF(s)ds$ and simplifying, we obtain that:

$$\frac{\int_{x}^{\infty} PDF(s+\Delta x)ds}{\int_{x}^{\infty} PDF(s)ds} < \frac{\int_{x}^{\infty} PDF(s)R(x, \Delta x)ds}{\int_{x}^{\infty} PDF(s)ds} = R(x, \Delta x)\frac{\int_{x}^{\infty} PDF(s)ds}{\int_{x}^{\infty} PDF(s)ds} = R(x, \Delta x),$$

which is the condition required by Equation S1 for $p_{\Delta x}(x)$ to be increasing. Thus, if $\frac{PDF(x+\Delta x)}{PDF(x)}$ is strictly decreasing in x, $p_{\Delta x}(x)$ is indeed strictly increasing in x.

S2.3. Necessary condition for $\boldsymbol{p_{\Delta x}(x)}$ to be increasing in $\boldsymbol{x}$ for all $\boldsymbol{\Delta x}$.

We claim that for $p_{\Delta x}(x)$ to be increasing in x for all Δx, the CDF must grow exponentially to 1 as $x \to \infty$. Thus, if the CDF doesn't grow exponentially to 1 as $x \to \infty$, we can immediately conclude that the process reflected by x cannot constitute an hourglass process for all choices of Δx. We show this in two steps: (1) we show that for $p_{\Delta x}(x)$ to increase with x for all Δx, the hazard function corresponding to the CDF:

$$h(x) = \frac{CDF'(x)}{1 - CDF(x)} \tag{S2}$$

must be increasing as well, then (2) we show that for the hazard function to be increasing, the CDF must grow exponentially to 1 as $x \to \infty$.

S2.4. For $\boldsymbol{p_{\Delta x}(x)}$ to increase with $\boldsymbol{x}$, the hazard function must be increasing

Suppose $p_{\Delta x}(x)$ is increasing in x for all x and for any Δx. Then, for any $x_2 > x_1$ and for any Δx,

$$\frac{CDF(x_2 + \Delta x) - CDF(x_2)}{1 - CDF(x_2)} > \frac{CDF(x_1 + \Delta x) - CDF(x_1)}{1 - CDF(x_1)}.$$

Thus,

$$\frac{\frac{CDF(x_2+\Delta x)-CDF(x_2)}{\Delta x}}{1 - CDF(x_2)} > \frac{\frac{CDF(x_1+\Delta x)-CDF(x_1)}{\Delta x}}{1 - CDF(x_1)}.$$

Because both sides of the preceding expression must be non-negative, it follows from the squeeze theorem that,

$$\lim_{\Delta x \to 0} \frac{\frac{CDF(x_2+\Delta x)-CDF(x_2)}{\Delta x}}{1 - CDF(x_2)} \geq \lim_{\Delta x \to 0} \frac{\frac{CDF(x_1+\Delta x)-CDF(x_1)}{\Delta x}}{1 - CDF(x_1)}.$$

That is,

$$\frac{CDF'(x_2)}{1 - CDF(x_2)} \geq \frac{CDF'(x_1)}{1 - CDF(x_1)},$$

so

$$h(x_2) \geq h(x_1),$$

implying that the hazard function is non-strictly increasing. However, the only case where $h(x_2) = h(x_1)$ and h is non-strictly increasing would be where h is constant on $[x_1, x_2]$. In that case, as shown below, $1 - CDF(x)$ would be exponential, which means the corresponding PDF would be an exponential distribution. Hence, $p_{\Delta x}(x)$ would be constant. Indeed, for exponential distributions, the CDF is:

$$CDF(x) = 1 - e^{-x}.$$

Using this CDF in the definition of the REM propensity measure and simplifying yields that for any Δx:

$$\begin{aligned} p_{\Delta x}(x) &= \left[\left(1 - e^{-(x+\Delta x)}\right) - \left(1 - e^{-x}\right)\right] / \left[1 - \left(1 - e^{-x}\right)\right] \\ &= \left[e^{-x} - e^{-x-\Delta x}\right] / \left[e^{-x}\right] \\ &= 1 - e^{-\Delta x}, \end{aligned}$$

a constant. This would however, contradict that $p_{\Delta x}(x)$ is increasing. Thus, it must actually be the case that

$$h(x_2) > h(x_1). \ \square$$

S2.5. For the hazard function to be increasing, the CDF must grow exponentially to 1 as $\boldsymbol{x \to \infty}$. Note that it is possible to uniquely identify a distribution to a hazard function. In particular,

$$\begin{aligned} h(x) &= \frac{CDF'(x)}{1 - CDF(x)} \\ &= \frac{d}{dx}\left[-\log(1 - CDF(x))\right]. \end{aligned}$$

Thus, for any x small enough that the CDF has not yet reached 1,

$$-\log(1 - CDF(x)) = c + \int h(x)dx,$$

and so for any x_0,

$$1 - CDF(x) = (1 - CDF(x_0))e^{-\int_{x_0}^{x} h(s)ds}.$$

However, if h is increasing, then if $x > x_0$, we get that $h(x) > h(x_0)$ and

$$\begin{aligned} 1 - CDF(x) &< (1 - CDF(x_0))e^{-\int_{x_0}^{x} h(x_0)ds} \\ &= (1 - CDF(x_0))e^{-h(x_0)(x-x_0)}. \end{aligned}$$

That is, if h is increasing, $1 - CDF(x) \sim O\left(e^{-h(x_0)x}\right)$ as $x \to \infty$. So as $x \to \infty$, $CDF(x)$ "grows exponentially to 1". $\square$

S3. KEY PROPERTIES OF THE REM PROPENSITY MEASURE $p_{\Delta|N|}(|N|)$

S3.1. Proof that $\boldsymbol{p_{\Delta|N|}(|N|)}$ based on a log-GMM model approaches 0 as $|\boldsymbol{N}| \to \infty$.

Our proposed REM propensity measure $p_{\Delta|N|}(|N|)$ is computed from a Gaussian mixture model fit to the PDF of $\log(|N|)$, where $|N|$ is the amount of NREM sleep accumulated since the previous REM bout. Fitting $\log(|N|)$ to a Gaussian mixture model is equivalent to fitting $|N|$ to a sum of two lognormal distributions. Here we show that any propensity $p_{\Delta x}(x)$ computed from a log-normal distribution for x approaches 0 as $x \to \infty$, contrary to the assumed condition that $p_{\Delta x}(x)$ must be increasing for an hourglass process discussed in Section S. To do this, it suffices to investigate the limit for an individual lognormal distribution.

To show this limit, we first simplify the condition for $\lim_{x\to\infty} p_{\Delta x}$ for general distributions. In particular, we note that both the numerator and denominator in the definition of the propensity measure tend to 0 as $x \to \infty$. Assuming that the CDF of the accumulating quantity underlying propensity is differentiable, we apply l'Hopital's rule to the limit:

$$\begin{aligned}\lim_{x\to\infty} p_{\Delta x}(x) &= \lim_{x\to\infty} \frac{CDF'(x+\Delta x) - CDF'(x)}{-CDF'(x)} \\ &= 1 - \lim_{x\to\infty} \frac{CDF'(x+\Delta x)}{CDF'(x)}.\end{aligned}$$

Rewriting in terms of the corresponding PDF yields that

$$\lim_{x\to\infty} p_{\Delta x}(x) = 1 - \lim_{x\to\infty} \frac{PDF(x+\Delta x)}{PDF(x)}. \tag{S3}$$

To find $\lim_{x\to\infty} p_{\Delta x}(x)$ specifically for a lognormal distribution, we simplify Eq. S3 applied to a lognormal distribution:

$$\begin{aligned}\lim_{x\to\infty} \frac{PDF(x+\Delta x)}{PDF(x)} &= \lim_{x\to\infty} \frac{\frac{1}{(x+\Delta x)\sigma\sqrt{2\pi}} e^{-\frac{1}{2}(\frac{\log(x+\Delta x)-\mu}{\sigma})^2}}{\frac{1}{x\sigma\sqrt{2\pi}} e^{-\frac{1}{2}(\frac{\log(x)-\mu}{\sigma})^2}} \\ &= \lim_{x\to\infty} \frac{x}{(x+\Delta x)} e^{-\frac{1}{2}(\frac{\log(x+\Delta x)-\mu}{\sigma})^2 + \frac{1}{2}(\frac{\log x-\mu}{\sigma})^2} \\ &= \lim_{x\to\infty} \frac{x}{(x+\Delta x)} e^{-\frac{1}{2}(\frac{((\log(x+\Delta x))^2 - 2\mu\log(x+\Delta x)) - (\log x)^2 + 2\mu \log x}{\sigma^2})} \\ &= \lim_{x\to\infty} \frac{x}{(x+\Delta x)} e^{-\frac{1}{2}(\frac{(\log((x+\Delta x)x) - 2\mu)\log(\frac{x+\Delta x}{x})}{\sigma^2})}.\end{aligned}$$

Continuing to simplify the limit:

$$\begin{aligned}\lim_{x\to\infty}\frac{PDF(x+\Delta x)}{PDF(x)} &= \lim_{x\to\infty}\frac{x}{(x+\Delta x)}e^{-\frac{(\log((x+\Delta x)x)-2\mu)}{2\sigma^2}\log(\frac{x+\Delta x}{x})} \\ &= \lim_{x\to\infty}\frac{x}{(x+\Delta x)}e^{\log\left((\frac{x+\Delta x}{x})^{-\frac{(\log((x+\Delta x)x)-2\mu)}{2\sigma^2}}\right)} \\ &= \lim_{x\to\infty}\frac{x}{(x+\Delta x)}\left(\frac{x+\Delta x}{x}\right)^{-\frac{(\log((x+\Delta x)x)-2\mu)}{2\sigma^2}} \\ &= \lim_{x\to\infty}\left(\frac{x+\Delta x}{x}\right)^{-1-(\frac{\log((x+\Delta x)x)-2\mu}{2\sigma^2})}.\end{aligned}$$

We now show that the preceding limit is 1, thus establishing that $p_{\Delta x}(x)\to 0$ by using Eq. S3. Let

$$y=\left(\frac{x+\Delta x}{x}\right)^{-1-(\frac{\log((x+\Delta x)x)-2\mu}{2\sigma^2})}.$$

To show that $y\to 1$, it suffices to show that $\log(y)\to 0$. Indeed, simplifying $\log(y)$:

$$\begin{aligned}\lim_{x\to\infty}\log y &= \lim_{x\to\infty}\left(-1-\frac{\log((x+\Delta x)x)-2\mu}{2\sigma^2}\right)\log\left(\frac{x+\Delta x}{x}\right) \\ &= \lim_{x\to\infty}\left[-1-\frac{\log((x+\Delta x)x)-2\mu}{2\sigma^2}\right]\Big/\left[\left(\log\left(\frac{x+\Delta x}{x}\right)\right)^{-1}\right],\end{aligned}$$

putting us in a position to apply l'Hopital's rule:

$$\begin{aligned}\lim_{x\to\infty}\log y &= \lim_{x\to\infty}\left[-\frac{(2x+\Delta x)}{2\sigma^2(x+\Delta x)x}\right]\Big/\left[-\frac{\left(\log\left(\frac{x+\Delta x}{x}\right)\right)^{-2}\left(1-\frac{\Delta x}{x^2}\right)}{\frac{x+\Delta x}{x}}\right] \\ &= \frac{1}{2\sigma^2}\lim_{x\to\infty}\frac{(2x+\Delta x)\left(\log\left(\frac{x+\Delta x}{x}\right)\right)^2}{x^2\left(1-\frac{\Delta x}{x^2}\right)} \\ &= \frac{1}{2\sigma^2}\lim_{x\to\infty}\frac{\left(2+\frac{\Delta x}{x}\right)\left(\log\left(1+\frac{\Delta x}{x}\right)\right)^2}{x\left(1-\frac{\Delta x}{x^2}\right)} \\ &= 0. \square\end{aligned}$$

S3.2. The propensity $\boldsymbol{p_{\Delta|N|}(|N|)}$ based on a log-GMM model has a local maximum

Since $p_{\Delta|N|}(|N|)$ is non-negative and approaches 0 as $|N|\to\infty$, $p_{\Delta|N|}(|N|)$ will have a local maximum if it is increasing for some $|N|$ (assuming $p_{\Delta|N|}(|N|)$ is sufficiently smooth).

To show that the propensity is increasing for some $|N|$ and therefore has a local maximum, we differentiate $p_{\Delta|N|}(|N|)$ with respect to $|N|$ and write $CDF'(|N|)$ in terms of the corresponding PDF, $\mathbb{P}(|N|)$:

$$p'_{\Delta|N|}(|N|) = \frac{\big[\mathbb{P}(|N|+\Delta|N|) - \mathbb{P}(|N|)\big]\big[1-CDF(|N|)\big] - \big[CDF(|N|+\Delta|N|) - CDF(|N|)\big]\big[-\mathbb{P}(|N|)\big]}{\big[1-CDF(|N|)\big]^2}$$
$$= \frac{\mathbb{P}(|N|+\Delta|N|) - \mathbb{P}(|N|+\Delta|N|)CDF(|N|) - \mathbb{P}(|N|) + CDF(|N|+\Delta|N|)\mathbb{P}(|N|)}{\big[1-CDF(|N|)\big]^2}.$$

Thus, if $\mathbb{P}(|N|) < \mathbb{P}(|N|+\Delta|N|)$ for some $|N|$, then at that $|N|$, the propensity is increasing since

$$\frac{\mathbb{P}(|N|+\Delta|N|)}{\mathbb{P}(|N|)} > 1,$$

and since the CDF is an increasing function,

$$1 > \frac{1-CDF(|N|+\Delta|N|)}{1-CDF(|N|)},$$

implying that

$$\frac{\mathbb{P}(|N|+\Delta|N|)}{\mathbb{P}(|N|)} > \frac{1-CDF(|N|+\Delta|N|)}{1-CDF(|N|)},$$

which after cross-multiplying yields the desired result:

$$\mathbb{P}(|N|+\Delta|N|) - \mathbb{P}(|N|+\Delta|N|)CDF(|N|) - \mathbb{P}(|N|) + CDF(|N|+\Delta|N|)\mathbb{P}(|N|) > 0.$$

To show that the propensity $p_{\Delta|N|}(|N|)$ based on a log-GMM model has a local maximum, all that remains to be shown is that there is some choice of $|N| = |N|_0 > 0$ that makes $\mathbb{P}(|N|_0) < \mathbb{P}(|N|_0 + \Delta|N|)$. However, there may not be any $|N|$ making $\mathbb{P}(|N|) < \mathbb{P}(|N|+\Delta|N|)$ unless $\Delta|N|$ is small enough. Specifically, we claim that if $\Delta|N|$ is small enough, it is possible to find an interval $[0, \Delta|N| + \epsilon)$ over which the PDF of the log-GMM is strictly increasing for some constant $\epsilon > 0$, and thus there exists some sufficiently small $|N|_0$ such that $(|N|_0, |N|_0 + \Delta|N|) \subseteq [0, \Delta|N| + \epsilon)$, making the desired condition that $\mathbb{P}(|N|_0) < \mathbb{P}(|N|_0 + \Delta|N|)$ be true. In particular, the PDF of a log-GMM model is increasing at least until $|N|$ reaches the smallest of the maxima across the two log-normal distributions comprising it. Since the PDF of a lognormal distribution based on a normal distribution with mean μ and standard deviation σ has a maximum when $x = \exp(\mu - \sigma^2)$, the PDF of the log-GMM, given by

$$PDF(x) = k_l N_l(\log(x)) + (1-k_l)N_s(\log(x)),$$

must increase over the interval $x \in [0, \min\{\exp(\mu_l - \sigma_l^2), \exp(\mu_s - \sigma_s^2)\})$. Consequently, if

$$\Delta|N| < \min\{\exp(\mu_l - \sigma_l^2), \exp(\mu_s - \sigma_s^2)\},$$

there is some $\epsilon > 0$ so that the PDF of the log-GMM is increasing on $[0, \Delta|N| + \epsilon)$, so that the desired condition, $\mathbb{P}(|N|_0) < \mathbb{P}(|N|_0 + \Delta|N|)$, holds. □

S4. RELATIONSHIP WITH THE HAZARD FUNCTION

Our proposed REM propensity measure, for a stochastic quantity, X, is very similar to the corresponding hazard function (Eq. S2). Indeed, using the approximation that

$$CDF(x + \Delta X) - CDF(x) \approx CDF'(x)\Delta x,$$

we see that

$$h(x)\Delta x \approx \frac{CDF(x + \Delta x) - CDF(x)}{1 - CDF(x)},$$

which is the proposed REM propensity measure. That is,

$$p_{\Delta x}(x) \approx h(x)\Delta x. \tag{S4}$$

However, there are important differences between the proposed REM propensity measure and the hazard function.

S4.1. $\boldsymbol{h(x)\Delta x}$ sometimes poorly approximates $\boldsymbol{p_{\Delta x}(x)}$

If the PDF of the value of some accumulating quantity at the transition to REM is Gaussian, the hazard function fails to approximate the probability of entering REM sleep for large x. In fact, for any Δx, $h(x)\Delta x$ tends to ∞. However, $h(x)\Delta x$ is supposed to approximate the probability of entering REM sleep before accumulation of Δx units, and thus is bounded between 0 and 1. Therefore, using the hazard function to approximate $p_{\Delta x}$ introduces error of $O(x\Delta x)$. Indeed, if the PDF is a normal distribution with mean μ and standard deviation σ, i.e.,

$$PDF(x) = \frac{1}{\sigma\sqrt{2\pi}} e^{-0.5\left(\frac{x-\mu}{\sigma}\right)^2},$$

we have from l'Hopital's rule that

$$\begin{aligned}
\lim_{x\to\infty} h(x) &= \lim_{x\to\infty} \frac{CDF''(x)}{-CDF'(x)} \\
&= \lim_{x\to\infty} -\frac{PDF'(x)}{PDF(x)} \\
&= \lim_{x\to\infty} -\frac{-\left(\frac{x-\mu}{\sigma}\right)\frac{1}{\sigma\sqrt{2\pi}} e^{-0.5\left(\frac{x-\mu}{\sigma}\right)^2}}{\frac{1}{\sigma\sqrt{2\pi}} e^{-0.5\left(\frac{x-\mu}{\sigma}\right)^2}} \\
&= \lim_{x\to\infty} \frac{x-\mu}{\sigma} \\
&= \infty
\end{aligned}$$

In contrast, the probability $p_{\Delta x}(x)$ of entering REM before the accumulation of Δx units must by definition be between 0 and 1.

SUPPLEMENTARY FIGURES

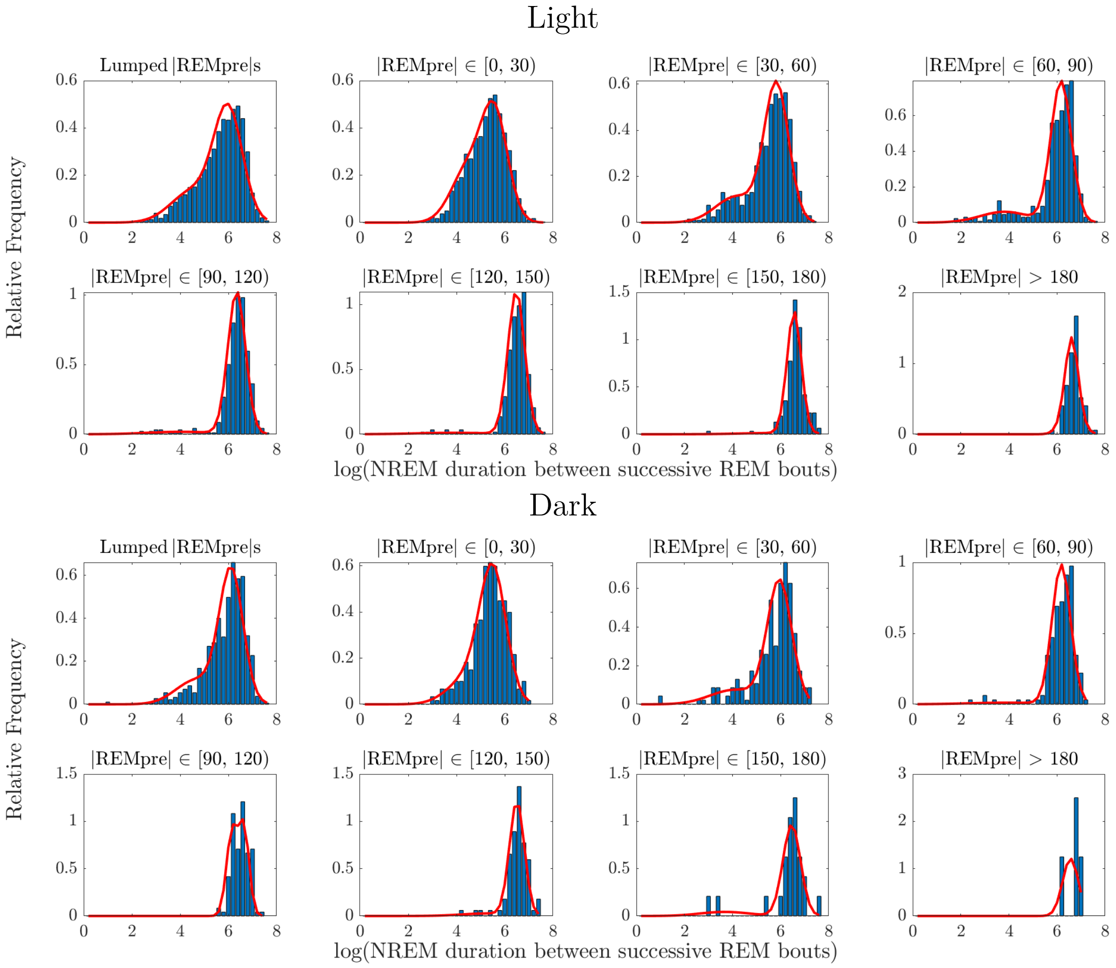

Figure S1: Empirical PDFs along with GMM fit curves for $\log(|N|)$ during the light and dark phases. The histograms depict the distributions of $\log(|N|)$ where $|N|$ is the total duration of NREM sleep occurring between successive bouts of REM sleep, within the lumped and binned datasets. The red curves show the GMM fit for the underlying probability distributions. Limited data are available for $|\text{REMpre}| > 180$, especially in data for the dark phase.

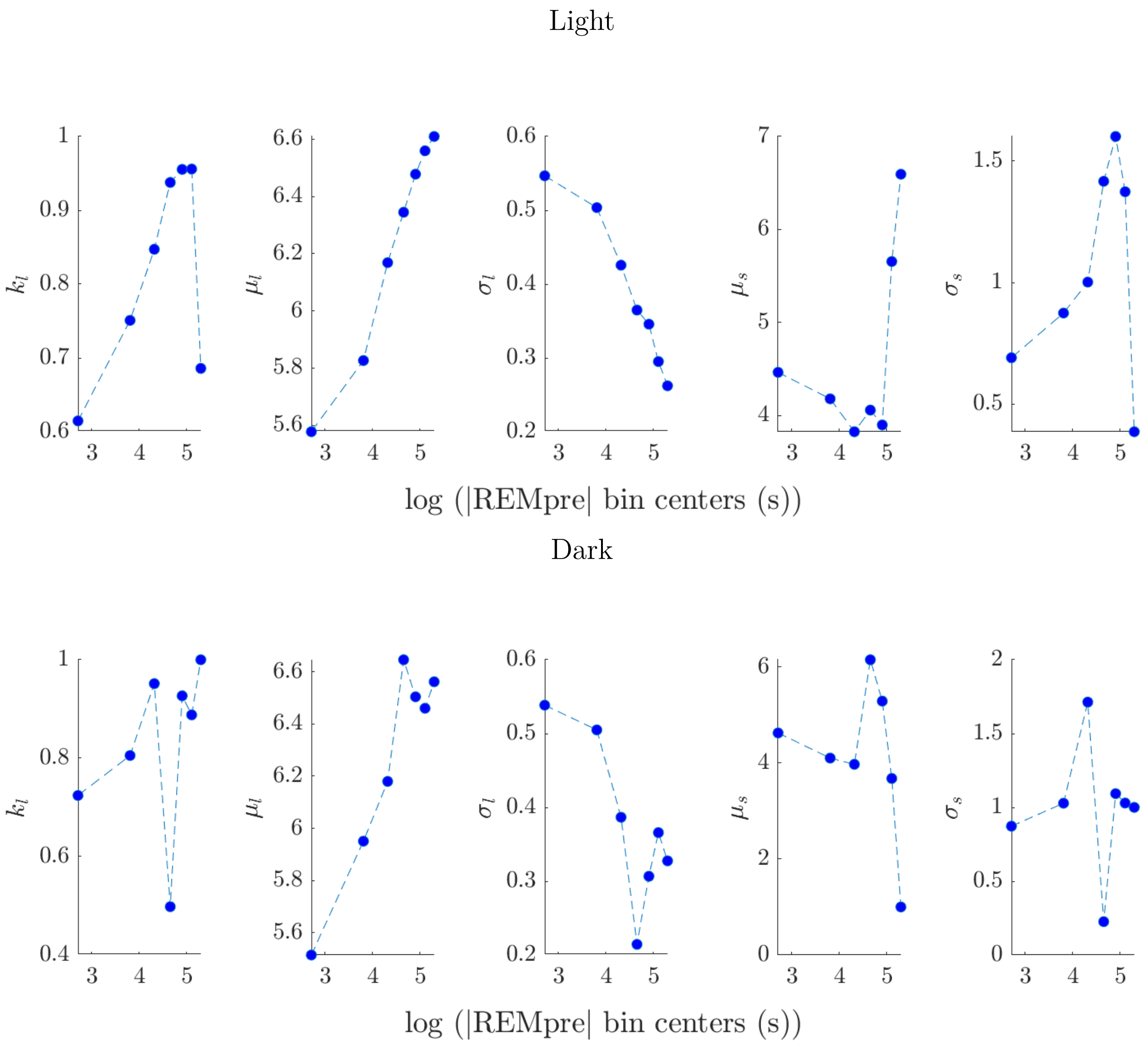

Figure S2: Parameter values for GMM fits to $\log |N|$ across |REMpre| bins in the light and dark phases. Each GMM fit is comprised of two Gaussians: N_l and N_s, where N_l has a longer mean than N_s. The parameter $k_l \in [0, 1]$ is the weighting towards the Gaussian with the higher mean; μ_l and σ_l are the mean and standard deviation of the Gaussian with the longer mean; and μ_s and σ_s are the mean and standard deviation of the Gaussian with the shorter mean. The parameter k_l generally exhibits an increasing trend, indicating that the GMM has more weight towards the larger mean as the log (|REMpre| bin centers (s)) increases. However, for e.g., the light phase when log (|REMpre| bin centers (s)) is at its largest, the means of the shorter and longer distributions are nearly equal, and hence are weighted nearly equally, explaining why k_l drops from around 0.95 to around 0.7.

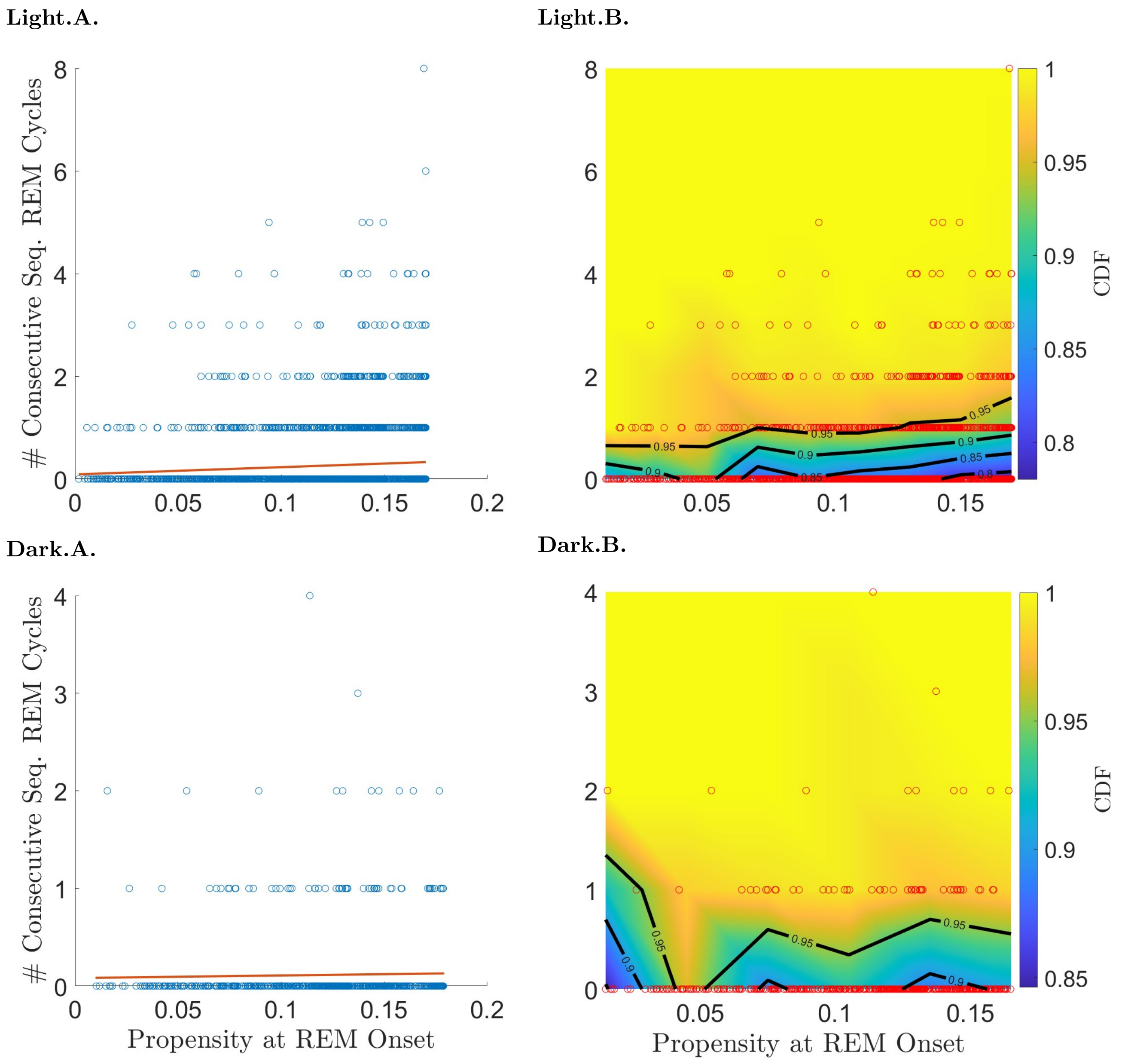

Figure S3: Relationship between pre-peak REM propensity and the number of sequential cycles that follow. We consider REM cycles that occur before the peak propensity. Rows 1 and 2 describe the relationship between the REM propensity at the end of a single cycle and the number of consecutive sequential REM cycles that follow, for the light and dark phases, respectively. Columns 1 - 2, from left to right, show, respectively, scatter plots (blue circles) of the number of sequential REMs (if any) that follow a single cycle vs REM propensity along with the line of best fit (red line) as calculated by linear regression; and CDFs (heat-color) and the corresponding contours (black lines) for the number of consecutive sequential REMs (if any) that follow, binned according to REM propensity, with the scatter plot data (red circles) overlaid. Notably, before the peak in propensity and only during the light phase, there is a statistically significant relationship between propensity and the number of sequential REM cycles that follow a single REM cycle.

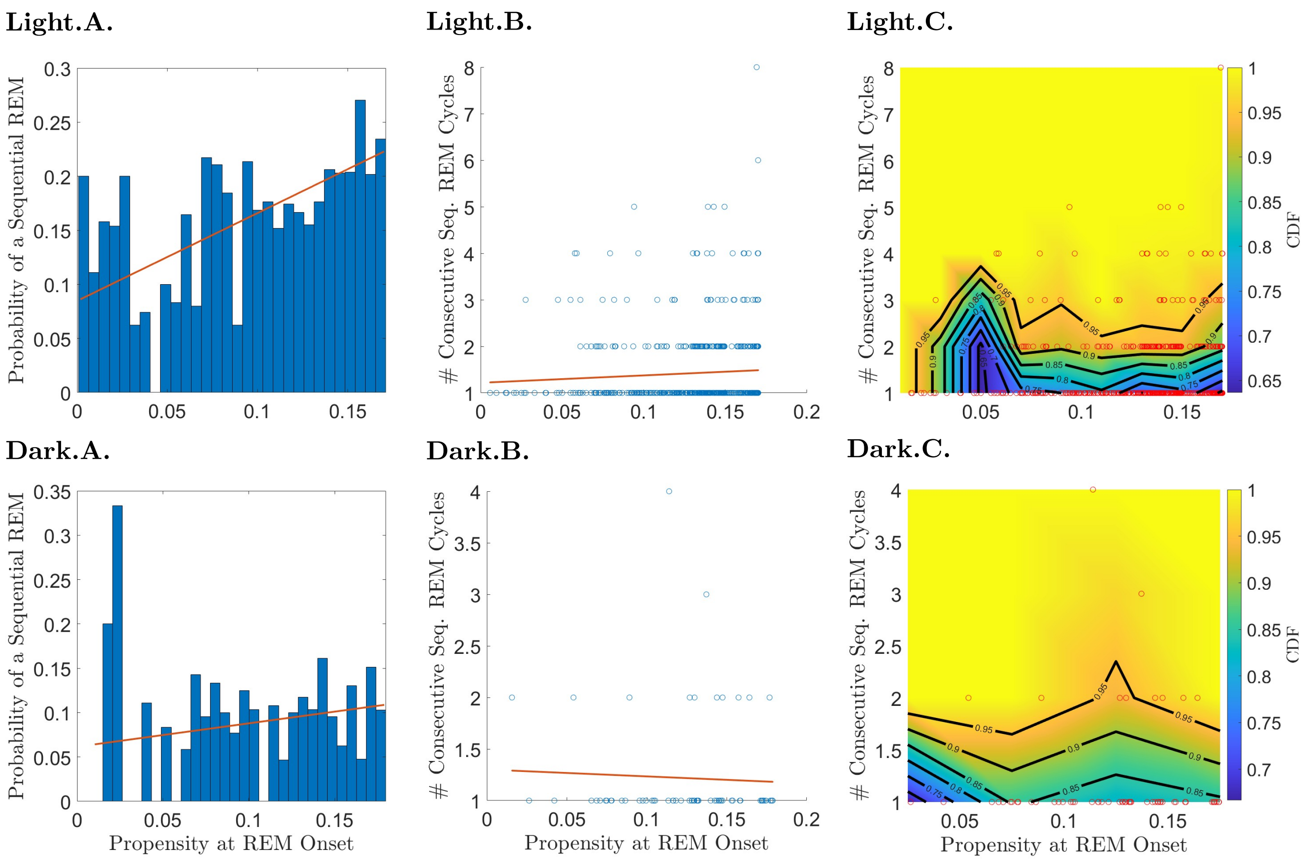

Figure S4: Relationship between pre-peak REM propensity and the length of subsequent sequential chains vs the probability that a sequential chain occurs. We consider REM cycles that occur before the peak propensity. Rows 1 and 2 describe the relationship between the REM propensity at the end of a single cycle and the chain of consecutive sequential REM cycles that follows (if present), for the light and dark phases, respectively. The leftmost column displays a bar chart of the probability that a sequential REM bout follows a single REM bout across bins of REM propensities at the onset of REM sleep at the end of the single cycle. The red best-fit line shows a positive (and statistically significant) slope during the light phase, indicating that the probability of having a sequential REM increases with REM propensity. Columns 2-3, from left to right, show, respectively, scatter plots (blue circles) of REM propensity vs the number of sequential REMs that follow a single REM cycle given that at least one sequential REM cycle follows along with the line of best fit (red line) as calculated by linear regression; and CDFs (heatmap color) and the corresponding contours (black lines) for the number of sequential REM cycles binned according to REM propensity, with the scatter plot data (red circles) overlaid. Before the peak in propensity and for both the light and dark phases, there is no statistically significant correlation between propensity and the number of sequential REM cycles that follow given that at least one sequential REM cycle follows.

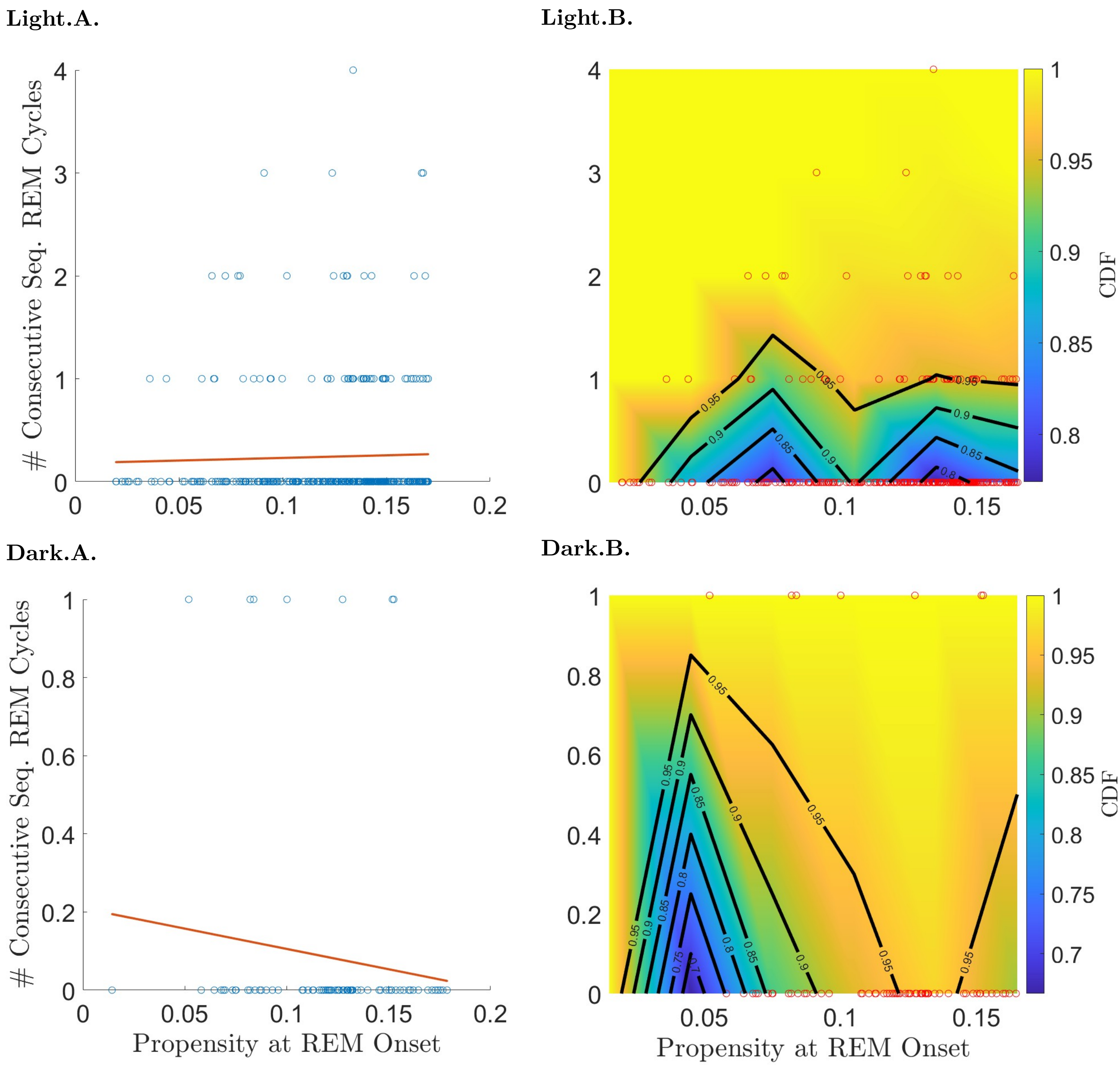

Figure S5: Relationship between post-peak REM propensity and the number of sequential cycles that follow. We consider REM propensity after it peaks. Rows 1 and 2 describe the relationship between the REM propensity at the end of a single cycle and the number of consecutive sequential REM cycles that follow, for the light and dark phases, respectively. Columns 1 - 2, from left to right, show, respectively, scatter plots (blue circles) of the number of sequential REMs (if any) that follow a single cycle vs REM propensity along with the line of best fit (red line) as calculated by linear regression; and CDFs (heat-color) and the corresponding contours (black lines) for the number of consecutive sequential REMs (if any) that follow, binned according to REM propensity, with the scatter plot data (red circles) overlaid. Notably, following the peak in propensity, there are no statistically significant relationships between propensity and the number of sequential REM cycles that follow a single REM cycle.

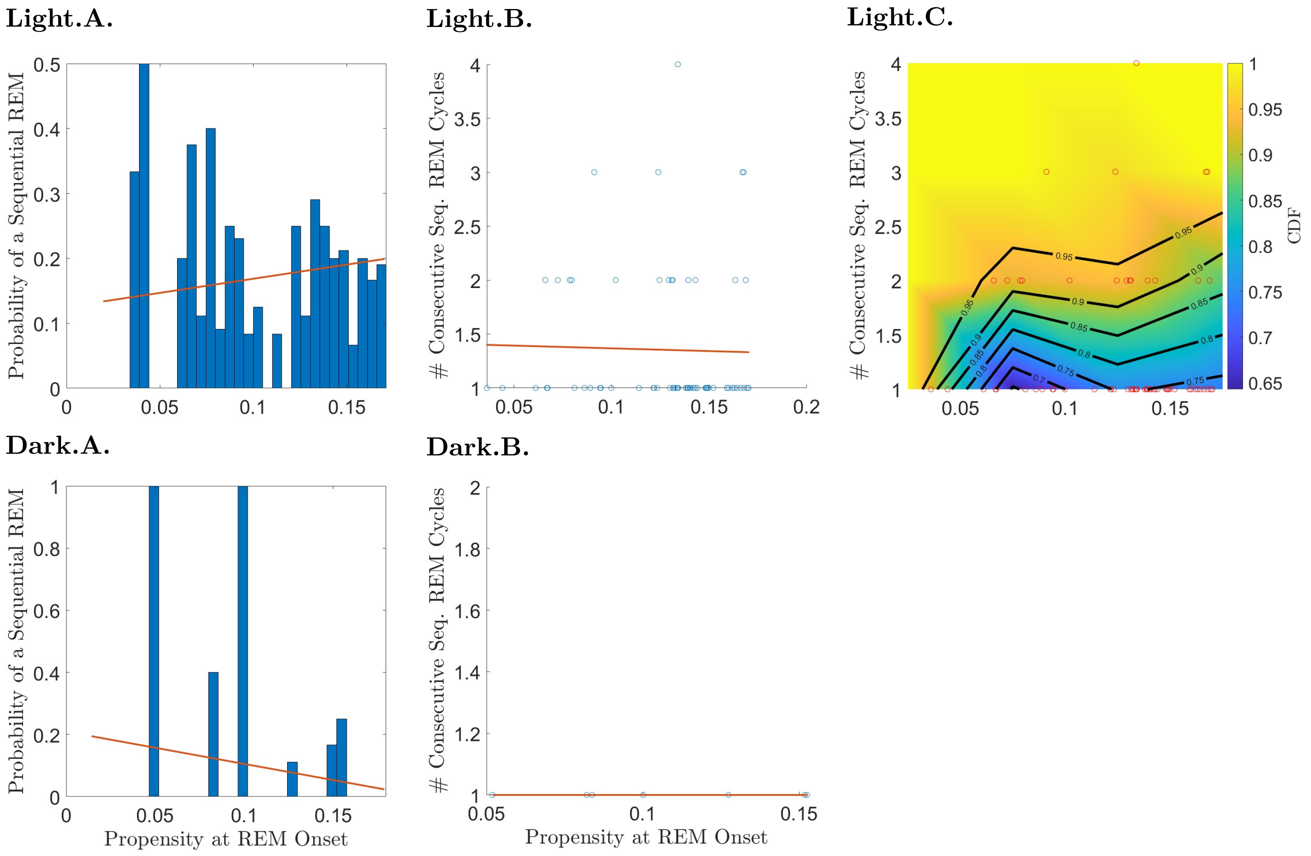

Figure S6: Relationship between post-peak REM propensity and the length of subsequent sequential chains vs the probability that a sequential chain occurs. We consider REM propensity after it peaks. Rows 1 and 2 describe the relationship between the REM propensity at the end of a single cycle and the chain of consecutive sequential REM cycles that follows (if present), for the light and dark phases, respectively. The leftmost column displays a bar chart of the probability that a sequential REM bout follows a single REM bout across bins of REM propensities at the onset of REM sleep at the end of the single cycle. The red best-fit line shows slight (and non-statistically-significant) slopes during the light and dark phases, indicating that the probability of having a sequential REM does not have a clear relationship with REM propensity. Columns 2-3, from left to right, show, respectively, scatter plots (blue circles) of REM propensity vs the number of sequential REMs that follow a single REM cycle given that at least one sequential REM cycle follows along with the line of best fit (red line) as calculated by linear regression; and CDFs (heatmap color) and the corresponding contours (black lines) for the number of sequential REM cycles binned according to REM propensity, with the scatter plot data (red circles) overlaid. The heatmap corresponding to the dark phase is absent because all single cycles that are followed by a sequential cycle are followed by only one sequential cycle, and thus the heatmap would add no information to the scatter plot from panel (Dark.B.). After the peak in propensity, there are no clear relationships between propensity and the number of sequential REM cycles that follow given that at least one sequential REM cycle follows.
